# Machine learning reveals mesenchymal breast carcinoma cell adaptation in response to matrix stiffness

**DOI:** 10.1371/journal.pcbi.1009193

**Published:** 2021-07-23

**Authors:** Vlada S. Rozova, Ayad G. Anwer, Anna E. Guller, Hamidreza Aboulkheyr Es, Zahra Khabir, Anastasiya I. Sokolova, Maxim U. Gavrilov, Ewa M. Goldys, Majid Ebrahimi Warkiani, Jean Paul Thiery, Andrei V. Zvyagin

**Affiliations:** 1 ARC Centre of Excellence for Nanoscale Biophotonics, Macquarie University, Sydney, Australia; 2 Institute for Biology and Biomedicine, Lobachevsky State University, Nizhny Novgorod, Russia; 3 Graduate School of Biomedical Engineering, University of New South Wales, Sydney, Australia; 4 Institute for Regenerative Medicine, Sechenov University, Moscow, Russia; 5 School of Biomedical Engineering, University of Technology Sydney, Sydney, Australia; 6 Centre of Biomedical Engineering, Sechenov University, Moscow, Russia; 7 Laboratory of Medical Nanotechnologies, Federal Biomedical Agency, Moscow, Russia; 8 Bioland Laboratory, Guangzhou Regenerative Medicine and Health, Guangdong Laboratory, Guangzhou, China; 9 Institute of Bioorganic Chemistry of the Russian Academy of Sciences, Moscow, Russia; Weizmann Institute of Science, ISRAEL

## Abstract

Epithelial-mesenchymal transition (EMT) and its reverse process, mesenchymal-epithelial transition (MET), are believed to play key roles in facilitating the metastatic cascade. Metastatic lesions often exhibit a similar epithelial-like state to that of the primary tumour, in particular, by forming carcinoma cell clusters via E-cadherin-mediated junctional complexes. However, the factors enabling mesenchymal-like micrometastatic cells to resume growth and reacquire an epithelial phenotype in the target organ microenvironment remain elusive. In this study, we developed a workflow using image-based cell profiling and machine learning to examine morphological, contextual and molecular states of individual breast carcinoma cells (MDA-MB-231). MDA-MB-231 heterogeneous response to the host organ microenvironment was modelled by substrates with controllable stiffness varying from 0.2kPa (soft tissues) to 64kPa (bone tissues). We identified 3 distinct morphological cell types (morphs) varying from compact round-shaped to flattened irregular-shaped cells with lamellipodia, predominantly populating 2-kPa and >16kPa substrates, respectively. These observations were accompanied by significant changes in E-cadherin and vimentin expression. Furthermore, we demonstrate that the bone-mimicking substrate (64kPa) induced multicellular cluster formation accompanied by E-cadherin cell surface localisation. MDA-MB-231 cells responded to different substrate stiffness by morphological adaptation, changes in proliferation rate and cytoskeleton markers, and cluster formation on bone-mimicking substrate. Our results suggest that the stiffest microenvironment can induce MET.

## Author summary

The epithelial-mesenchymal transition (EMT), a process by which epithelial cells lose their cell polarity and cell-cell adhesion and gain migratory and invasive properties to become mesenchymal cells, is believed to play the key role in the initiation of the metastatic cascade. The ability of mesenchymal cells to survive, transform and establish colonies in distant organs is well known but not well understood. To gain insight into this process, we developed a workflow using machine learning and image analysis of cells to examine how morphology (or physical form and structure) and biochemical properties of individual (triple negative) breast carcinoma cells vary in response to their interaction with a substrate of variable stiffness. We found that mesenchymal breast cancer cells responded to different substrate stiffness by morphological adaptation, changes in proliferation rate and cytoskeleton markers such as cell-cell adhesion protein E-cadherin. Surprisingly, the cell properties on the stiffest tested substrate that mimicked bone stiffness changed dramatically–they formed multicellular clusters with distinct E-cadherin localisation at the cell-cell adhesion surfaces. Our results suggest that the stiffest microenvironment can induce cell transitioning from mesenchymal to epithelial cell phenotype.

## Introduction

Breast carcinoma, one of the most commonly occurring cancers in women with over 2 million new cases diagnosed in 2018 [1], primarily spread to the liver, bones, lungs and brain [2]. About 15% of all invasive breast cancers classified as triple negative breast carcinoma (TNBC) are characterised by the lack of estrogen and progesterone receptors and HER2 amplification. TNBC are not responsive to current endocrine and other targeted therapies limiting the treatment options to surgery, chemotherapy, and radiation [3]. It has been shown that TNBC patients have a higher probability of recurrence and distant relapse during the first five years compared to other types of breast cancer [4] and shorter post-recurrence survival [5]. Therefore, understanding the underlying mechanisms of TNBC metastasis is crucial to improve the treatment outcomes.

One of the viable hypotheses relevant to TNBC metastasis relate to organotropic metastasis and is originated from the Paget’s “seed and soil” hypothesis [6,7]. It states that certain cancer types prefer to grow in certain organs in a way that cannot be explained by the circulatory patterns alone. Indeed, a solid body of evidence suggests that metastatic events are regulated by many factors including tumour-intrinsic properties, specific characteristics of the host organ microenvironment, and their interaction [8]. Among TNBC-related cell lines, MDA-MB-231 is recognised as a highly mesenchymal invasive cell phenotype. Notably, unlike the general cohort of TNBC, MDA-MB-231 have the propensity to form osteolytic bone metastases in vivo when inoculated into the bloodstream of immunodeficient mice [9].

It is recognised that one of the key processes facilitating the metastatic progression of carcinoma cells is the epithelial-mesenchymal transition (EMT). During this process, a progressive loss of intercellular junctional complexes is initiated in the polarised epithelial cells accompanied by the acquisition of a mesenchymal phenotype through the remodelling of the actin microfilament network, weaker intercellular adhesion and a number of other mesenchymal markers [10–12]. Hence, EMT triggers carcinoma cells dissociation from the primary tumour, invasion through the surrounding stroma, intravasation into the lymphatic or blood vessels and dissemination to distant body sites.

Despite its lethal effects, metastasis is a highly inefficient process [13]. Following extravasation from the bloodstream and infiltration of the parenchyma of a distant organ, disseminated tumour cells, either individually or in clusters, are exposed to an unfamiliar microenvironment characterised by a drastically different structural and molecular composition. A critical gap in our understanding of cancer metastasis, therefore, lies between the initial interaction of disseminated tumour cells with the host organ microenvironment and their successful outgrowth into micrometastases.

Carcinoma cells with an activated EMT program can revert to the epithelial phenotype through mesenchymal-epithelial transition (MET) allowing them to establish new colonies after reaching their destination in secondary sites. Cellular and non-cellular components of both primary tumour and host organ microenvironment play crucial roles in the initiation and progression of each step of the metastatic process including EMT [12,14,15] and MET [16–18]. Among the non-cellular components, the properties of the extracellular matrix (ECM), including stiffness, structure, and composition showed a significant impact on the carcinoma cell plasticity through modulation of mechanotransduction [19,20]. A number of recent studies have recognised stiffness of the ECM as one of the critical mechanical factors contributing to the promotion of EMT in various types of cancer [21–24].

Despite numerous studies, the EMT-MET hypothesis fails to explain colonisation of secondary organs by carcinoma cells which are quasi unable to engage in MET. MDA-MB-231 cell line displaying a pronounced mesenchymal phenotype has been shown to form metastatic lesions in vivo [25]. We have found that the MDA-MB-231 colonisation patterns in whole-organ decellularized liver scaffolds varied dramatically across loose parenchymal (Young modulus, ~2 kPa) and denser (Young modulus, >2 kPa) stromal scaffold compartments [26], where cell morphology was one of the discriminants of the colonisation patterns. Cells infiltrated rapidly the parenchymal tissue, and their dominant morphological trait was identified as small round shape cells, whereas spindle-shaped, large-footprint cells were prevalent in the stromal compartment. Besides, the intriguing propensity for clustering of MDA-MB-231 cells on the stromal substrates was observed.

In this study, we have investigated the heterogeneous response of carcinoma cells to substrates fabricated with physiologically relevant stiffness levels. We deliberately chose essentially mesenchymal MDA-MB-231 cell phenotype to investigate the mechanisms enabling the formation of micrometastases by cells quite refractory to change their phenotype via MET.

It has been recently shown that cancer cell morphology is correlated with tumorigenic and metastatic potentials in vivo providing an easily measurable quantity [27]. Therefore, we use morphological profiling as a surrogate endpoint to measure the effect of stiffness on TNBC cell behaviour. We investigated the coupling between biochemical and morphological features using immunocytochemistry and examined the cell clustering mechanism, which may be a pivotal process in forming micrometastases.

Rapidly progressing state-of-the-art approaches of analysing and interpreting large biological datasets, including single-cell measurements derived from optical cell images, provide meaningful and oftentimes unexpected insight into both physical and molecular states of an individual cell. These approaches allow capturing cell heterogeneity in comparison with the existing population-based profiling [28,29]. In line with recent publications, in our study, we aimed to demonstrate a more data-driven approach by adopting a single-cell resolution technique [27,30]. Indeed, algorithms of machine learning are capable of compiling a large number of parameters and extract relationships between different factors to capture patterns invisible to the naked eye.

In this study, we report on the evaluation of the cellular adaptations to substrates of varying stiffness at the single-cell level. Using multi-parametric image-based cell profiling [31], followed by the application of machine learning techniques, we have identified subpopulations with distinct morphological cell types (morphs) and demonstrated the relationship between stiffness and the prevailing morphology. Further, we have analysed the expression levels of several biomarkers conventionally associated with epithelial and mesenchymal phenotypes to uncover the underlying molecular alterations mediated by substrate stiffness. Taken together, our results suggest that the softer microenvironment, especially at the 2-kPa stiffness level, promotes more aggressive morphs of MDA-MB-231 cells. It was intriguing to find that the substrate, with the stiffness of 64 kPa corresponding to that of the bone tissue, promoted the expression of E-cadherin inducing pronounced cell clustering.

## Results

### Cell adhesion to extracellular matrix components

**Fig 1:**
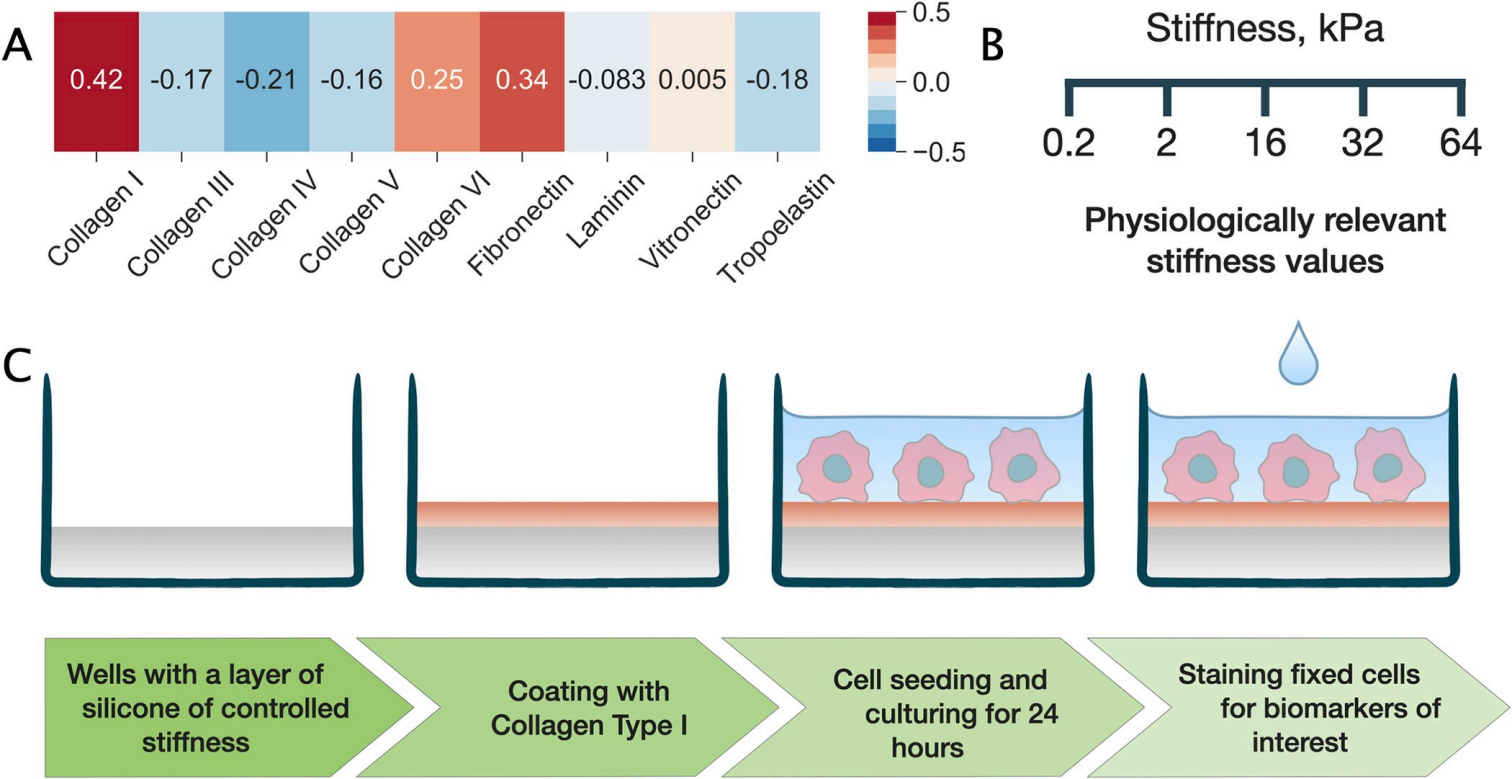
Choice of ECM protein coating and experimental design. (a) A heat map shows the values of Pearson’s correlation coefficient measuring the association between ECM protein concentration and the number of cells attached. (b) Substrate stiffness values investigated in this study. (c) In the designed experiment, MDA-MB-231 cells were seeded on silicone substrates of different stiffness coated with collagen type I. Afterwards, cells were cultured for 24 h, fixed, stained for biomarkers of interest and imaged using confocal microscopy.

In our study, we used a representative human TNBC cell line MDA-MB-231 to investigate morphological and molecular changes in response to substrate stiffness. First, to uncouple the chemically-mediated effects of the ECM, we explored how the biochemical composition of the underlying substrate affects cell adhesion. We simultaneously tested a total of 36 conditions consisting of the 9 most common ECM proteins taken in various concentrations and combinations (see Materials and Methods). Each condition was replicated in 9 spots imprinted on a hydrogel surface and the non-fouling material of the rest of the slide ensured specific cellular attachment. As a proxy for cell adhesion, we counted the number of cells attached to each spot 24 hours after seeding (Table B and Fig B in S1 Text). Collagens 1, 6, fibronectin and vitronectin appeared to facilitate cell adhesion while adhesion to collagens 3,4,5, laminin and tropoelastin was very minimal (Table B in S1 Text). Correlation analysis was performed to determine whether, for any of the ECM proteins, there were any associations between its concentration and the number of attached MDA-MB-231 cells. Fig 1A demonstrates that collagen I and fibronectin concentrations had the highest correlation with the number of adherent cells. Conversely, the higher concentrations of collagen type III, IV, V and tropoelastin were associated with decreased numbers of attached cells. Therefore, we chose collagen type I for further investigations.

### Multi-parametric image-based cell profiling

Evaluation of the stiffness-induced morphological and molecular changes in TNBC cells was performed in several steps. First, TNBC cells were cultured on substrates with five elastic moduli varying from 0.2 kPa to 64 kPa (Fig 1B). Routinely used tissue culture plates typically have a stiffness of 2–4 GPa, a multitude of times more rigid than tissues in the human body [32,33]. Silicon inserts, on the other hand, allow mimicking physiologically relevant conditions. Our choice of stiffness values was governed by the rigidity profiles of organs and tissues in which carcinoma cells reside following their dissemination [32,34]. The biocompatible silicone substrates placed on the bottom of wells were coated with collagen type I prior to cell seeding as schematically illustrated in Fig 1C. TNBC cells seeded in equal densities were cultured for 24 hours followed by fixation and immunostaining.

**Fig 2:**
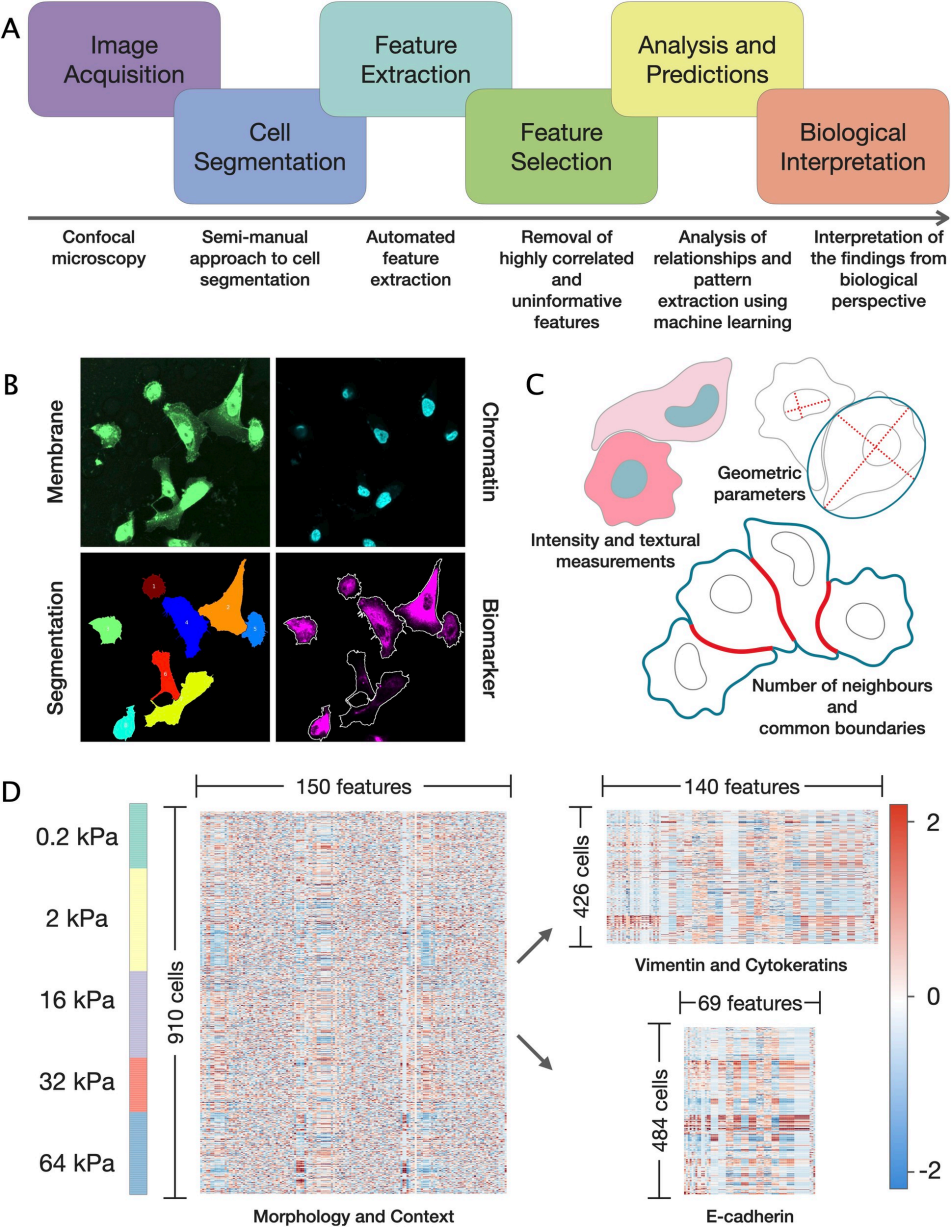
Generation of single-cell profiles and data analysis workflow. (a) A flow chart illustrating the key steps of image-based cell profiling and data analysis workflow. (b) Schematic representation of the image segmentation process: cells were labelled with WGA and DAPI to visualise cytoplasm and nuclei. Extracted cell outlines were used to quantify the intensity of E-cadherin, vimentin or cytokeratins within each cell. (c) Illustrations of the measurements calculated for each cell: geometric parameters, intensity and texture of the fluorescent signal, and measurements of the context. (d) Summary of the final dataset. Left column indicates the proportion of cells captured at each stiffness level. Morphological and contextual features of the cells were calculated (left); additionally, intensity and texture of the fluorescent biomarker signals were measured (right). Each row of the heatmaps corresponds to a single cell and each column represents a single feature. Z-score normalisation was applied to each feature to allow for direct comparison regardless of the scale. Right column indicates z-score values.

To quantify the effect of the stiffness at the single-cell level, we employed multi-parametric image-based profiling combined with the application of statistical analysis and machine learning techniques. The key steps of the data analysis workflow are shown in Fig 2A. To visualise cell membranes and nuclei and evaluate the expression of the biomarkers in each cell, cells were specifically immunolabelled with fluorescent dyes and imaged using confocal microscopy. We divided each of the 5 plates equally and stained one half for vimentin and pan-cytokeratin, and the other half for E-cadherin which allowed us to limit the number of fluorophores per well to 4 and avoid overlapping signals (see Materials and Methods).

Image segmentation was performed to outline cells and nuclei and use the extracted boundaries for quantification of the fluorescent signal intensity from E-cadherin or vimentin and cytokeratin within each cell as well as obtaining various geometric and contextual measurements (Fig 2B and 2C, Fig A and B in S2 Text). Fig 2D provides a comprehensive summary of the final dataset. For each of the 910 cells, we calculated 150 features describing cell morphology and context (Fig 2D, left). These include cell and nucleus shape descriptors such as the area, circularity and nuclear-cytoplasmic ratio (NCR); measurements of the shape irregularity such as solidity and compactness; measurements of the cell polarity calculated as a distance between the centroids of a cell and its nucleus; and contextual measurements such as the number of neighbouring cells and the proportion of the shared boundary. Upon visual examination, some cells cultured in the most rigid conditions appeared to form multicellular clusters (84 cells) and thus were excluded from the main dataset (826 cells remained) and considered separately.

### The effect of stiffness on TNBC cell density and morphology

**Fig 3:**
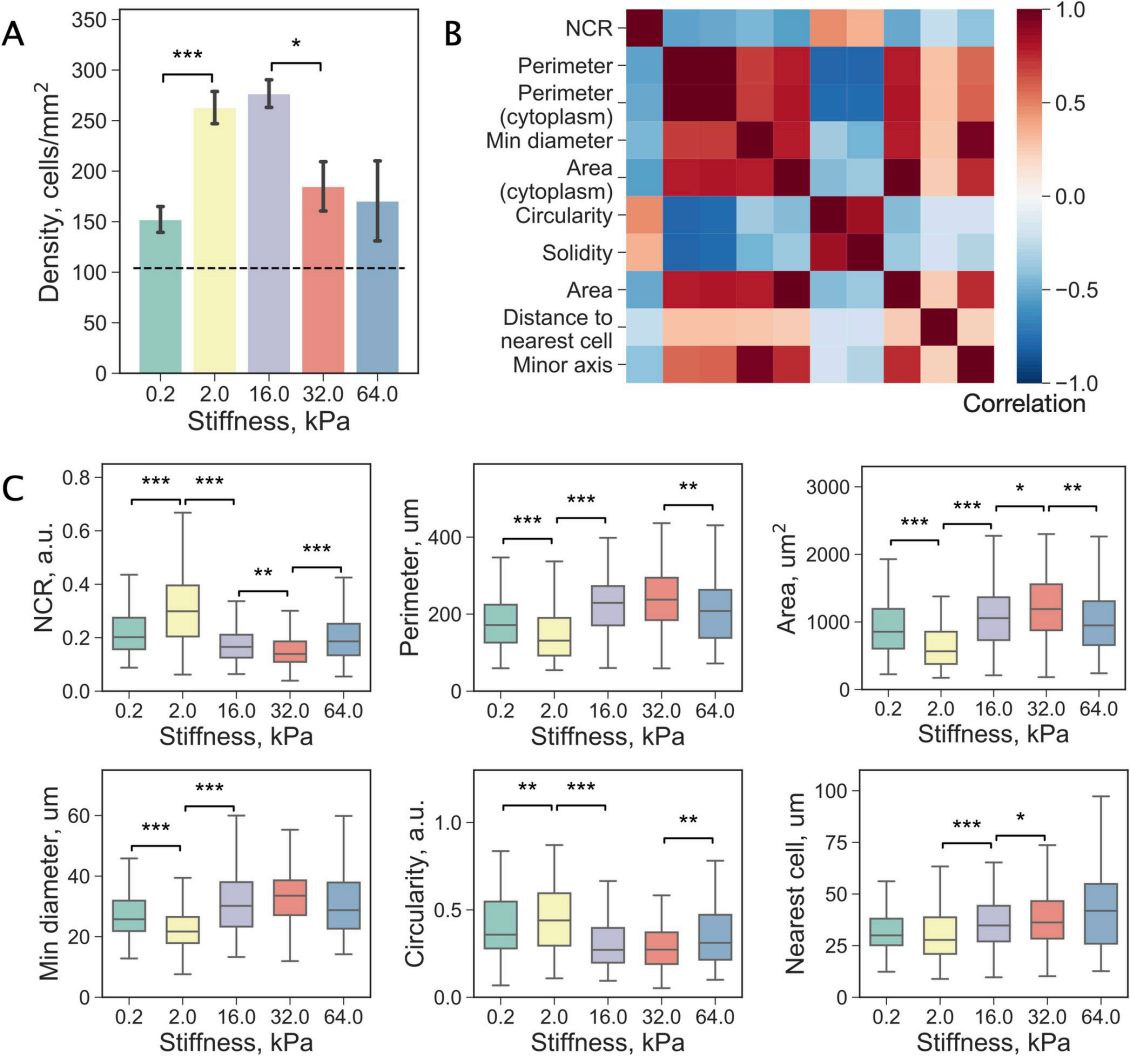
Distinct adaptations to substrate stiffness by TNBC cells. (a) An increase in cell density compared to baseline (dashed line) after cells were cultured for 24 hours on substrates with different stiffness values, n = 4 replicates per stiffness value. (b) Analysis of the association between the top 10 parameters modulated by substrate stiffness. Most measurements are related and thus are highly correlated with each other. (c) Changes in the distributions of NCR, cell perimeter, area, smallest diameter, circularity, and distance to the nearest cell in response to substrate stiffness. Distributions are reported using box plots: a box shows the median value, first and third quartiles, whiskers indicate median +/-1.5 * IQR. Significance of the difference assessed by Welch’s t-test (‘***’: p < 0.001, ‘**’: p < 0.01, ‘*’: p < 0.05), n = number of cells, see Table A in S2 Text.

We first sought to evaluate differences in the cell densities versus substrate stiffness. Images at low magnification were acquired to capture the fluorescent signal from cell chromatin located in the central region of each well (Fig C in S2 Text). Cells were initially seeded at equal densities and cultured for 24 hours followed by fixation. We observed significantly higher cell densities at 2 and 16 kPa indicating that these stiffness levels positively affect either cell attachment or proliferation, or both (Fig 3A).

Next, we analysed the 150 features describing the individual cell morphology and its relationship with surrounding cells. The effect of the substrate stiffness was immediately evident from the average profiling (Fig A in S3 Text); however, a single cell-based approach was further employed to exploit the intrinsic heterogeneity of the TNBC cell population. Analysis of Spearman’s rank correlation coefficients (*r_s_*) revealed several cell characteristics associated with the substrate stiffness value (Fig B in S3 Text). Since Spearman’s correlation is not robust to non-monotonic relationships, we verified these results using two other approaches (see Materials and Methods, Table A in S3 Text). The list of the top 10 parameters that displayed the strongest correlation with the substrate stiffness is given in Table B in S3 Text along with feature descriptions. While none of the parameters showed a strong association with substrate stiffness, all three methods indicated the presence of some sort of dependence.

It is important to note that some of these measurements are not independent of each other as can be seen from the correlation matrix shown in Fig 3B, for example, an increase in the cell area naturally results in extension of its perimeter. The effect of the stiffness on the value distributions of NCR, cell perimeter, area, smallest diameter, circularity, and distance to the nearest cell is demonstrated in Fig 3C and Fig C in S3 Text. These variables were chosen as they provide complementary information about the system (pairwise |*r_s_*| < 0.8). Notably, NCR commonly used in histopathology as a predictor of malignancy [35] was found to have the highest absolute correlation coefficient (|*r_s_*| = 0.31). A local maximum can be seen at 2 kPa where cells displayed a wider range of NCR values with a higher median value. Conversely, the smallest NCR values were observed at 32 kPa with a positive trend towards the stiffest substrate (64 kPa).

### Matrix stiffness shifts the balance between TNBC cell morphs

**Fig 4:**
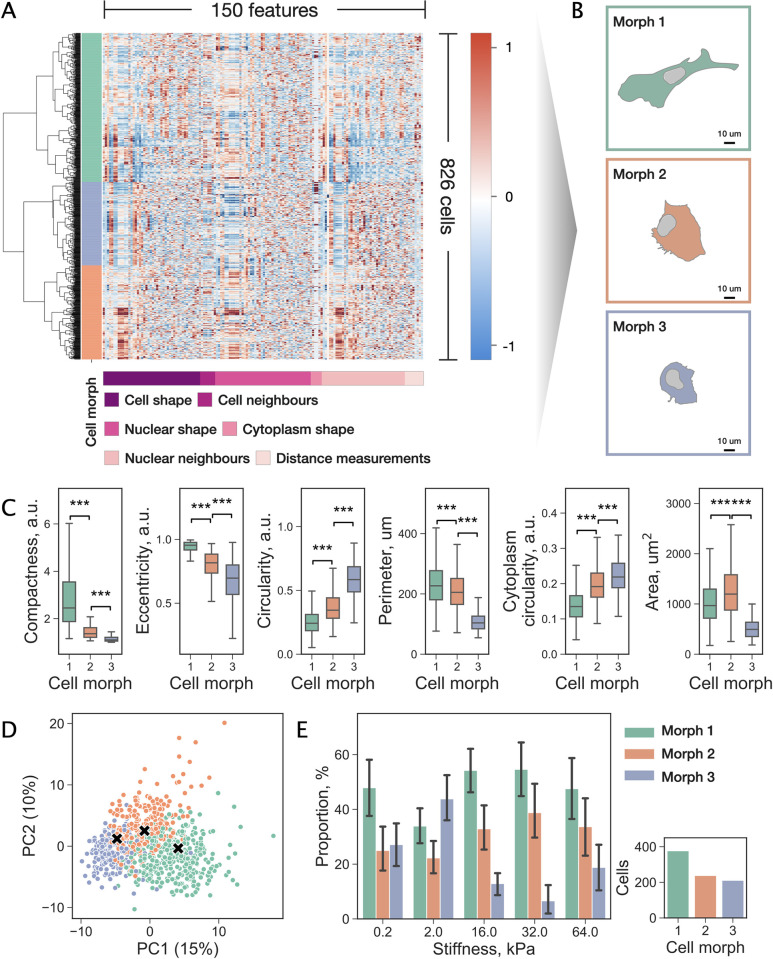
Identification of cell morphs using machine learning. (a) The results of HC applied to 826 cells based on similarities between single-cell profiles consisting of 150 morphological and contextual features. Left column indicates three cell morphs (green, blue, and red) identified in the total population of cells. Each row of the heatmap corresponds to a single cell and each column represents a single feature. Subgroups of features are depicted on the bottom. (b) Representative objects (medoids) of each of the three identified cell groups. (c) Top six features ranked by their contribution towards cluster separation. Distributions of the values are reported using box plots: a box shows the median value, first and third quartiles, whiskers indicate median +/-1.5 * IQR. Significance of the difference assessed by Welch’s t-test (‘***’: p < 0.001). (d) Projection of the data on the first two PCs and visualisation of the identified cell morphs. “×” markers indicate the centroids of the groups. (e) Proportion of cells of each morph across stiffness values. Bar plots represent average proportions, error bars indicate 95% confidence intervals, values calculated by grouping cells by images. The inset shows the total number of cells in each cluster.

The identification of distinct cell morphs was complicated by the fact that the analysed TNBC cells exhibited a continuum of morphological states. Dimensionality reduction techniques including Principal Component Analysis (PCA) revealed no apparent clusters suggesting a spectrum of closely related morphs across the cell population (Fig A in S4 Text). However, an unsupervised machine learning algorithm called hierarchical clustering (HC) enabled grouping of the data points (here, individual cells) into clusters (here, cell morphs) based on their profile similarity. As a result, we identified three subpopulations of cells displaying morphologies pertaining to distinct cell morphs. Our analysis was supported by two cluster validity indices (Silhouette score and Davies–Bouldin index) designed to select the number of clusters in the dataset (Fig B in S4 Text). Fig 4A demonstrated the results obtained by applying HC to single-cell profiles containing morphological and contextual measurements. To gauge the appearance of the cells, we identified representative objects for each cluster by calculating their medoids (Fig 4B). A medoid is defined as a data point with a minimum average distance to other objects in the cluster; therefore, it is conceptually similar to a centroid but restricted to members of the cluster.

**Fig 5:**
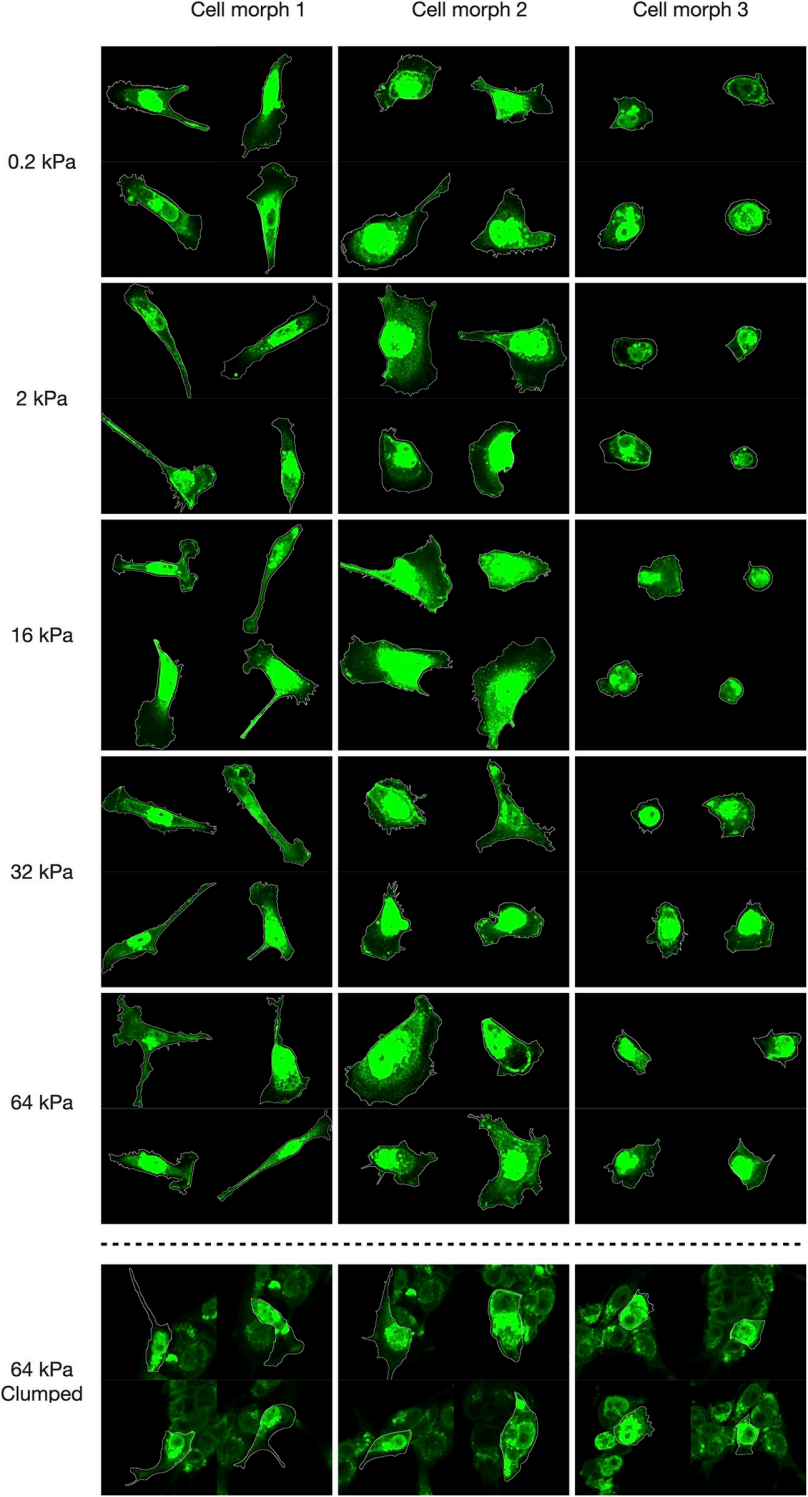
Visualisation of cell morphs. Representative images of cells cultured at different stiffness levels corresponding to the three cell morphs, as determined by the clustering algorithm. The bottom row provides examples of Clumped cells classified as one of the three cell morphs by the RF classifier. Cell membranes were visualised using WGA staining.

We then assessed the contribution of each feature towards cluster separability. The six most important features across all stiffness levels were compactness, eccentricity, circularity, perimeter, cytoplasm circularity, and area (Fig 4C, Fig C and D in S4 Text). The compactness describes the concavity of a membrane, the eccentricity relates to the elongation of a cell, and the circularity measures the similarity to a perfect circle. These characteristics alongside the perimeter and area differed significantly between the three cell morphs (p < 0.001). The projection of the data on the first two principal components (PCs) illustrates the inter-cluster spatial relationships (Fig 4D). These results together with the representative examples depicted in Fig 5 suggest that cell morphs 1, 2, and 3 display dramatically different appearances and are predominantly represented by irregular-shaped cells with lamellipodia, large flattened and compact roundish cells, respectively.

Interestingly, we observed considerable variation in the cell morph composition of cell populations across the substrate stiffness values (Fig 4E). Notably, all cell morphs exhibit local extrema at 2kPa–morphs 1 and 2 (green and red) have a local minimum, while morph 3 (blue) shows a local maximum. Small round cells (morph 3; blue) were prevailing at 2 kPa with a significant reduction in numbers on the stiffer substrates where cells were predominantly classified as morphs 1 and 2, i.e., stiffer substrates promote cell spreading and elongation with a prominent expression of lamellipodia (c.f. Fig 5). Note that the cell morph dependence versus the substrate stiffness falls out at 64 kPa–the expected trend of the morph 3 cell population decline versus the substrate stiffness is reversed, while the expected growth of the morph 1 and 2 cell population is not maintained. It is worth mentioning that the cell density depicted in Fig 3A did not appear to influence cell morphology as can be seen from the distributions of the number of cell neighbours and the fraction of the shared boundary (Fig E in S4 Text). When assessing the contribution of each feature to the cluster separability, we found that these parameters were not important, with the mutual information measuring 0.06 and 0.05, respectively. Taken together these results demonstrate a heterogeneous response to the matrix stiffness indicating significant changes in morphology and the composition of the cell population at 2 kPa and suggesting high morphological adaptability of TNBC cells.

### Expression of biomarkers is stiffness-dependent

To substantiate our observations, we assessed the role of the matrix stiffness on the expression of key biomarkers reporting on the cell cytoskeleton and cell-cell adhesion at the single-cell level. As described earlier and schematised in Fig 2D, about half of the total cell population was used to evaluate the expression of E-cadherin (Fig 2D, bottom right) while the other half was simultaneously stained for vimentin and cytokeratins (Fig 2D, top right).

**Fig 6:**
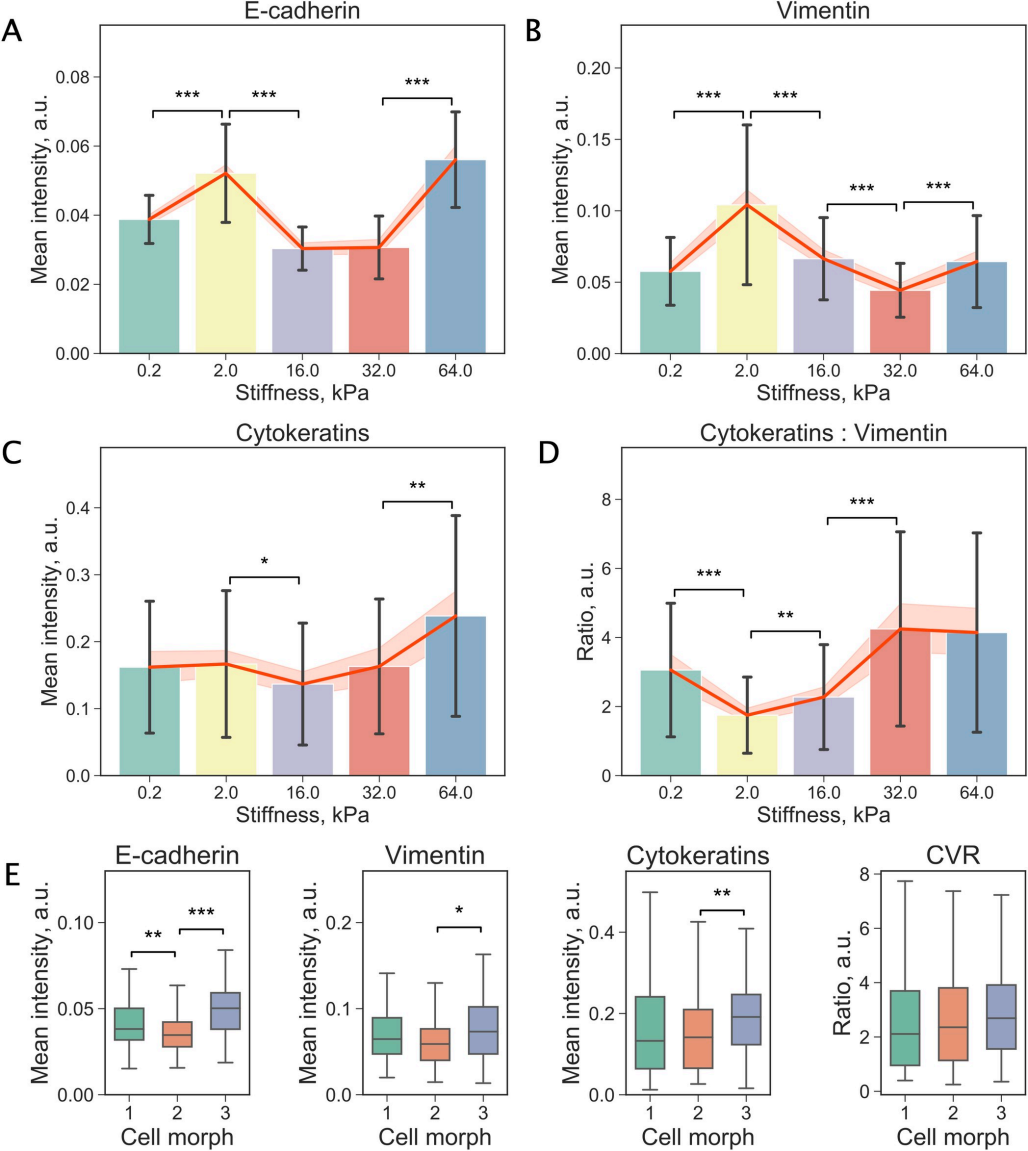
Changes in expression of biomarkers associated with epithelial and mesenchymal phenotypes. (a-c) Mean fluorescent signal from E-cadherin, vimentin, and cytokeratins, respectively, across substrates of different stiffness. (d) The ratio of fluorescence intensity of cytokeratins to vimentin (CVR) calculated for all stiffness values. For (a-d) values are mean, error bars indicate standard deviation. Orange line shows the mean values and the band indicates 95% confidence intervals. Significance of the difference assessed by Welch’s t-test (‘***’: p < 0.001, ‘**’: p < 0.01, ‘*’: p < 0.05), n = number of cells, see Table A in S2 Text. (e) Box plots comparing mean fluorescent signal from E-cadherin, cytokeratins, and vimentin in cells belonging to three cell morphs. There appears to be no difference in CVR between the three cell morphs as can be seen in the rightmost plot. Distributions of the values are reported using box plots: a box shows the median value, first and third quartiles, whiskers indicate median +/-1.5 * IQR. Significance of the difference assessed by Welch’s t-test (‘***’: p < 0.001, ‘**’: p < 0.01, ‘*’: p < 0.05).

Fig 6A demonstrates changes in the mean expression levels of E-cadherin across the substrate stiffness values. Two local maxima, at 2kPa and 64 kPa, are clearly observable. MDA-MB-231 cells also showed a peak in the expression of vimentin at 2kPa (Fig 6B). At the same time, the mean fluorescent intensity of cytokeratins increased gradually at 16–64 kPa (Fig 6C). We also calculated the ratio of the expression of cytokeratins to vimentin (CVR) and examined its distribution across stiffness values (Fig 6D) observing a nonlinear trend with a minimum at 2 kPa. It is worth noting that the expression of E-cadherin and vimentin at 2 kPa (and accordingly the CVR) differed significantly in comparison to the softest substrate (0.2 kPa) indicating that TNBC cells are highly sensitive to even small changes in matrix stiffness. Additionally, to investigate the variability of molecular distributions in response to substrate stiffness we performed PCA on the full sets of features describing the intensity and spatial distribution of E-cadherin, vimentin, and cytokeratins (Fig A in S5 Text). While no clearly separated groups could be observed, cells cultured on substrates with stiffness values 2.0 kPa and 32.0 kPa appear to reside on the opposing sides of the clusters in line with the trends that can be seen in Fig 6.

Our next goal was to relate the identified cell morphs to the expression levels of the biomarkers. Given that the integrated fluorescent signal is proportional to the area of a cell and that cell area was found to be one of the key characteristics distinguishing the clusters, we again examined fluorescent signal normalised by cell area (“mean intensity”). Notably, the mean intensity of E-cadherin in small round cells (morph 3; blue) was significantly higher than in its counterparts (Fig 6E). Contrary to our expectations, there was no difference in vimentin or in the CVR between the three cell morphs, as can be seen in Fig 6E. Overall, these results confirm the high sensitivity of MDA-MB-231 cells to substrate stiffness. The increase of both E-cadherin and vimentin (and the minimum in the CVR) at 2 kPa suggests possible events in MDA-MB-231 cells cultured on these substrates.

### Rigid substrates promote the formation of cell clusters

**Fig 7:**
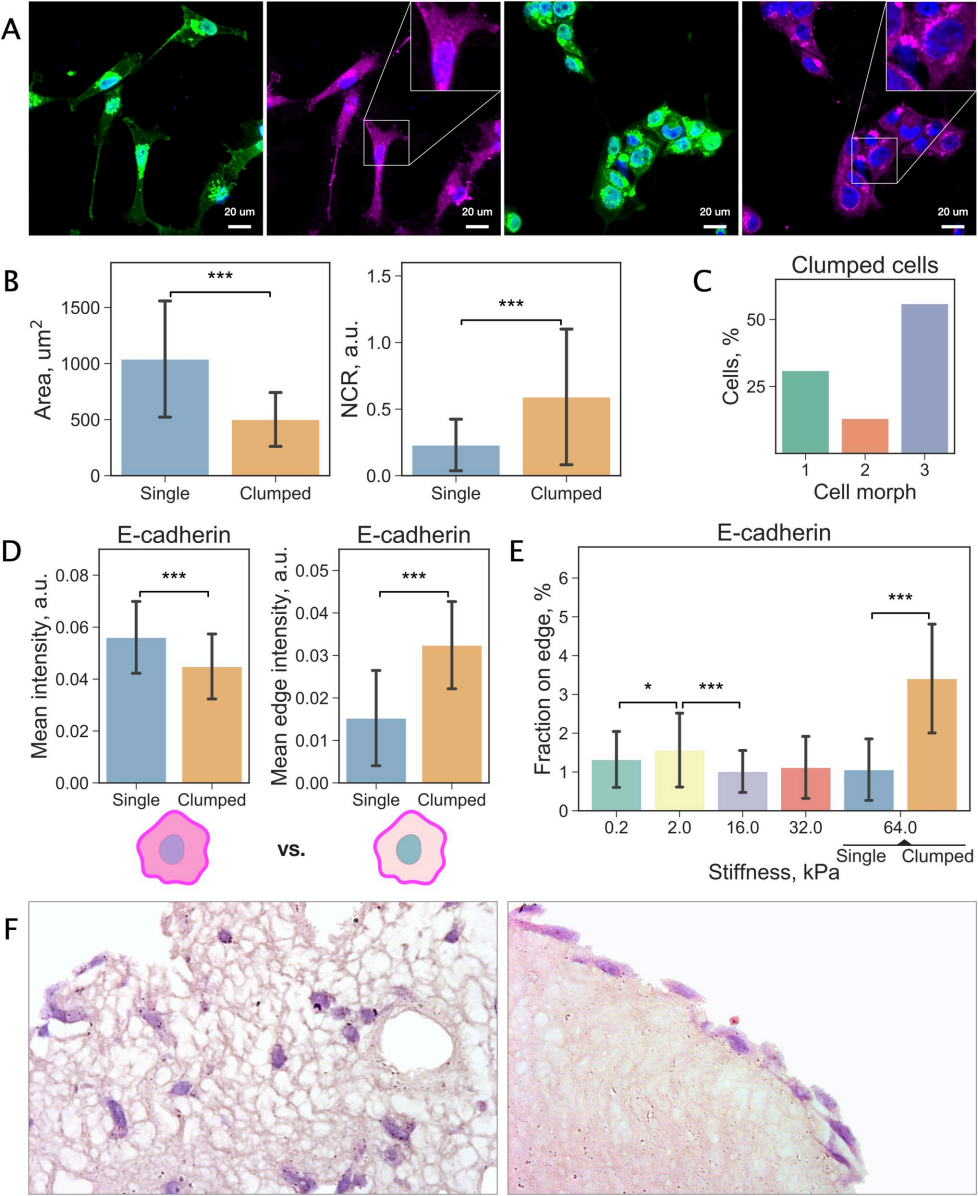
Comparison of individual and clustered cells cultured at 64 kPa. (a) Example images of individual and clumped cells cultured at 64 kPa. Cells stained with DAPI (blue) and WGA-FITC (green) to visualise cell nuclei and cytoplasm, and for E-cadherin (magenta). (b) Key differences between Single and Clumped cells. (c) Cell morph composition of Clumped cell population. (d) Mean intensity and mean edge intensity of E-cadherin in Single and Clumped cells. Schematic illustrations of the measurements are provided on the bottom. (e) Fraction of the total cell fluorescent signal located along the edge of a cell. Single and Clumped cells at 64 kPa are plotted separately. (f) TNBC cells (purple) displayed dramatically different morphological and colonisation patterns in the parenchymal (left) and stromal (right) compartments of our 3D liver tissue model. For (b), (d) and (e), values are mean, error bars indicate standard deviation. Significance of the difference assessed by Welch’s t-test (‘***’: p < 0.001, ‘**’: p < 0.01, ‘*’: p < 0.05).

A distinct tendency to multicellular clustering was observed on the most rigid substrates. Unlike other stiffness levels, where cells were growing individually, approximately half of the cells cultured at 64 kPa appeared to form tight cell clusters, indicating that the rigidity of a substrate can be a promoting factor. Representative images of individual and clustered cells (further referred to as “Single” and “Clumped”, respectively) are illustrated in Fig 7A. Clumped cells were excluded from the analysis above and examined separately (Fig A in S6 Text). Comparison of Single and Clumped cell populations cultured at 64 kPa revealed that, apart from obvious distinctions (Fig B in S6 Text), the two groups have significantly different NCR values due to differences in the total cell area (Fig 7B) while nuclear areas were comparable.

A Random Forest classification model was trained to classify cells into one of the cell morphs (morph 1, 2, or 3) and was validated on the test set achieving the F1 score of 0.98 (see Materials and Methods for details). The model was next used to estimate the distribution of the morphs in Clumped cells. As can be concluded from Fig 4D, among Single cells cultured at 64 kPa the majority exhibited irregular shapes (morph 1; green), which included relatively large cells with lamellipodial protrusions, as confirmed by visual examination. Conversely, morph 3 was prevailing in the population of Clumped cells (56%) while only a few cells assumed morph 2.

The analysis of the fluorescent signal distribution from E-cadherin revealed a peculiar pattern: while the mean intensity of E-cadherin was higher in Single cells, Clumped cells showed an increased localisation of E-cadherin on the cell surface suggesting a stronger capacity for the intercellular adhesion (Fig 7D). Additionally, we examined a fraction of the total cell fluorescent signal located along the edge of the cell. Fig 7E demonstrates the comparison of cells across all stiffness levels with values for Single and Clumped cells plotted separately. A pronounced difference in the fraction of cell surface-localised E-cadherin can be noted between Single and Clumped cells. Moreover, cells cultured on 2-kPa substrates also showed an increase in junctional E-cadherin. The latter observation once again suggests possible commonalities in cell behaviour between cells cultured on 2-kPa and 64-kPa substrates.

## Discussion

It is well established that the early steps of the metastatic cascade are facilitated by the epithelial-mesenchymal transition (EMT) allowing carcinoma cell detachment, migration, and dissemination. However, there is a lack of understanding of how cancer metastasis progresses from the initial dissemination of circulating tumour cells to a secondary organ and the ensuing formation of metastatic lesions [13]. Previous studies have pointed to the importance of the reverse process of EMT, namely, mesenchymal-epithelial transition (MET) for metastatic outgrowth, although this process remains poorly understood and unlike the inducers of EMT, few inducers of MET are known [25].

The expression of E-cadherin, a trait of epithelial cells, persists in many cancer types, including primary and metastatic tumours. Furthermore, E-cadherin junctional complexes are essential for several processes occurring during metastasis–collective cell migration and high-confluence cell proliferation [36]. The MB2 bone marrow tropic isogenic subline from MDA-MB-231 expresses E-cadherin in bone metastases, supporting the occurrence of MET induced by binding to E-selectin in the bone vascular niche [25], although these observations pointed towards a “non-canonical MET program” with unchanged RNA expression of the master transcriptional regulators of EMT, such as Snail1/2, Twist1/2 and Zeb1/2 suggesting a hybrid phenotype [37].

In light of this new evidence, we chose the cell line MDA-MB-231 characterised by the high mesenchymal index [38]. MDA-MB-231 cells are aggressive, treatment-resistant triple-negative breast carcinoma (TNBC) cells that are essentially mesenchymal since the epithelial biomarker E-cadherin expression is transcriptionally repressed by methylation of the E-cadherin promoter. We tested a set of physiologically relevant stiffness levels ranging from very soft (0.2 kPa) to highly rigid (64 kPa) and examined the heterogeneous response of the MDA-MB-231 cell population. Circulating tumour cells *in vivo* enter bone marrow by first residing for a while in the perivascular niche (2 kPa) where they partially regain the epithelial features. They later lodge into the bone matrix (64 kPa) and become osteolytic while shifting towards an epithelial-like phenotype.

We can make several comments on our results in the context of the recent observations outlined above. Firstly, our investigation of the adhesion of MDA-MB-231 cells to different components of the ECM aided by the correlation analysis suggest significant differences in the preferred and suppressed adhesion of these cells, where collagen type 1 displayed the largest adhesion. The ECM components that induce stronger adhesion can potentially contribute to cellular arrest in the tissue, while the components associated with decreased cell attachment can facilitate cell migration.

Secondly, we note that the cell density is the highest at 2 and 16 kPa, implicating the highest proliferation-to-apoptosis ratio, cell adhesion or both. It is possible that there exists a causal relationship between the cell density and cell morphology, for example, specific cell morphs may have a higher proliferation-to-apoptosis ratio. Conversely, higher cell density may result in smaller more rounded cells. Although we cannot rule out such dependency, our results add little support to this possibility.

Thirdly, by evaluating the profiles of individual cells, we showed that MDA-MB-231 cells, while displaying a continuum of morphologies, can be related to three distinct morphological types (termed cell morphs). Machine learning techniques allowed to identify the three subpopulations based on their geometric and contextual parameters and characterise each of these morphs by extracting the key distinguishing parameters. While a more granular partitioning can be produced by changing the cut-off in the dendrogram (Fig 4A), at the high level cells group into the identified three subpopulations. Taken together, our results show that morph 1 corresponds to cells with various irregularities and lamellipodial protrusions, morph 2 consists of large flattened cells, and morph 3 is represented by small compact round cells. Notably, in their recently published study, Devaraj and Bose report on the similar morphological states of MDA-MB-468 cells [39].

It is important to note that the displayed distinct morphological traits appeared highly variable across the substrate stiffness values. This implicates that cells respond to the substrate stiffness variation by remodelling their matrix adhesion and cytoskeleton. Notably, the ratios between the identified morphs change with an increase of the substrate stiffness in a nonlinear manner. Specifically, morph 3 dominated at 2 kPa but was significantly less present at the higher stiffness values. Interestingly, in a recent theoretical work, the authors showed that cells transition into one of the three cell morphs: weakly adhered (similar to morph 3), elongated with multiple protrusions (morph 1), or crescent-like (morph 2) depending on the strength of cell-substrate adhesion [40].

It is worth noting that single cell clones enriched with cells displaying a cell shape with a high aspect ratio akin to morph 1 do not show a high metastatic potential [27]. Both morphs 1 and 2 showed an increased cell population on the stiffer substrates. The morphological traits of an individual MDA-MB-231 cell have been reported to persist for several weeks. Single cell clones with the same morphological types displayed similar in vivo behavioural patterns, including tumorigenicity, survival in circulation, and metastatic potential [27]. In regard to cell motility, primarily flattened cells corresponding to morph 2 are expected to exert strong interaction with the substrate and are likely to be immotile due to the presence of large stable focal adhesions [41]. At the same time, protruding lamellipodia displayed by morph 1 is a hallmark of cell motility. Lastly, morph 3 may exhibit ameboid movements, however, video microscopy would be required to confirm this hypothesis.

Furthermore, we assessed the changes in expression levels of three biomarkers reporting on the cell cytoskeleton and intercellular adhesion aiming to investigate links between these changes and cell morphology. While the expression of cytokeratins was found to increase gradually with the stiffness, the intensity of vimentin displayed a nonlinear relationship peaking at the softer stiffness values. Interestingly, we also observed a local maximum in E-cadherin along with a peak in the vimentin expression at 2 kPa–this stiffness is typical for parenchymal type tissues e.g., liver and lungs. Mechanistic interpretation suggests that compliant substrates preclude the formation of widely spread cell-matrix junctions so that the cell retracts to a small round footprint. This hampered the cell motility and caused an upregulation of vimentin to restore this property typical for mesenchymal cells.

The stiffest tested substrate (64 kPa) typical for bone tissue induced dramatic variation in TNBC cell behaviour. We observed a subpopulation of MDA-MB-231 cells that formed multicellular clusters (“Clumped”). These cells appeared to have pronounced differences in both the morphology and expression of E-cadherin compared to the single cells cultured on the same substrate (“Single”). Notably, Clumped cells mostly displayed small round morphology contrary to the large irregular footprint prevalent among Single cells. In comparison with the overall cell population, the E-cadherin expression in Single cells cultured on 64 kPa substrate was significantly greater while in Clumped cells it was relatively low. However, the localisation of E-cadherin in Clumped cells was considerably shifted towards the cell surface. Previously, a similar trend in the E-cadherin signal localisation has been observed as a result of the maturation of the cell-cell E-cadherin-mediated contacts [42]. Moreover, it has been shown that as the cell-cell adhesion increases, cells become smaller and are characterised by a decrease in lamellipodia formation [43] thus providing a potential explanation for the morphological differences between Single and Clumped cells.

Predominant expression of E-cadherin at the cell-cell junctions speaks in favour of a stiffness-induced mechanism of cluster formation of mesenchymal cells in bone-type tissues. It is known that expression of E-cadherin alters cell morphology and decreases fibroblast-like migration in MDA-MB-231 cells [44]. Moreover, local microenvironment at the secondary site may induce re-expression of E-cadherin through the changes in epigenetic regulation of the E-cadherin gene in MDA-MB-231 [17]. Therefore, high extracellular matrix (ECM) stiffness might be one of the contributing–if not the key–factors forcing cell aggregation followed by re-expression of E-cadherin and changes in cell morphology, migratory and invasive behaviour [45]. However, while organ stiffness may play a role in bone metastasis it may not be a factor in other organs such as the liver, lung and brain which are also commonly colonised by breast carcinoma cells.

These results are also in line with the observations from our previous study, where we investigated colonisation of the liver parenchymal and stromal compartments by MDA-MB-231 cells [26]. We showed that the cell growth and invasion patterns, as well as their morphology, critically depend on the stiffness of the ECM. In particular, it was found that among rapidly proliferating cells deep into the soft (2 kPa) parenchymal tissue, the morphological trait of a small spheroidal shape dominated (Fig 7F, left). Meanwhile, the dense stromal substrate stimulated the morphological type characterised by elongation and a large footprint (Fig 7F, right). Finally, a tendency towards the formation of multicellular and multilayer clusters of MDA-MB-231 cells was also detected exclusively in the stromal compartments.

Overall, our work helps substantiate the evidence of a significant effect of the tumour microenvironment on the cancer cell morphological and molecular adaptability. The dramatic variation of the cell behaviour of the E-cadherin-mediated cluster formation occurred on the stiffest substrate pertinent to the bone tissue implicates a potentially new promoter for clustering of mesenchymal cells critically important in metastasis [46,47].

Further, it addresses the problem of intra-population heterogeneity and presents a framework for evaluating the morphological and molecular states of individual cells. We believe the proposed workflow can be adopted to analyse populations of cells cultured in various conditions and using a range of cell models as long as compatibility with confocal microscopy imaging and scaffold-free or gel-based 3D cultures is ensured.

It is worth mentioning, however, that the MDA-MB-231 cell line used in this study has been established a long time ago and over the years has been selected to a very stable highly mesenchymal metastatic phenotype. In the future, we aim to investigate whether differentiated breast cancer cells demonstrate a similar response to various substrate stiffness. Variable responses to EMT or MET inducers are observed using distinct cell lines and single-cell sequencing [48,49]. To further investigate this phenomenon, it would be necessary to analyse single cell-derived clones to determine which clones can form clusters. Furthermore, multicellular clusters observed at the most rigid substrate may have formed through other cadherins. Further experiments are required to verify these observations with cells stained for beta-catenin together with F-actin to show its localisation at the cell cortex. Finally, verification of the machine learning model on publicly available histological samples can illuminate the translational potential of this research, specifically, in the field of digital pathology.

## Materials and methods

### Ethics approval and consent to participate

No human or animal ethics approval was required for this study.

### Assay substrates

To choose the best ECM protein for coating, cells were seeded on an extracellular matrix screening array, ECM Select Array Kit Ultra-36 (Advanced Biomatrix, San Diego, USA), containing 36 ECM conditions on hydrogel surface. Each condition is a combination of 1 to 4 human-derived ECM proteins taken in a total concentration of 250 μg/mL. It is replicated in 9 spots each having a diameter of 400 μm (Fig A in S1 Text). The following ECM proteins were used: collagen I, collagen III, collagen IV, collagen V, collagen VI, fibronectin, laminin, vitronectin, tropoelastin (Table A in S1 Text). The array was washed and prepared for cell seeding according to the manufacturer’s instructions.

To study the effect of stiffness, cells were seeded into CytoSoft Imaging 24-well plates (Advanced Biomatrix) containing a thin silicon layer of different stiffness on the bottom of each well. The elastic moduli of silicon were 0.2 kPa, 2 kPa, 16 kPa, 32 kPa, and 64 kPa. Glass-bottom 24-well plates (Ibidi, Gräfelfing, Germany) were used for control experiments and treated according to the same protocols unless stated otherwise. Plate wells were coated with human type I collagen solution of 3 mg/mL, VitroCol (Advanced Biomatrix). Collagen was diluted in 1x DPBS (15 μg/mL), and 1 mL of solution was dispensed into each well to thoroughly coat the surface. Coated plates were incubated at room temperature, covered for 1 hour. After incubation, coated surfaces were rinsed two times with PBS.

### Cell culture

MDA-MB-231 (ECACC 92020424) breast cancer cells were subcultured and maintained in complete culture medium prepared from Dulbecco’s Modified Eagle’s Medium/Nutrient Mixture F-12 Ham (DMEM/F-12; Sigma-Aldrich) supplemented with 10% fetal bovine serum (FBS; Sigma-Aldrich, Sent-Luis, USA) and 1% penicillin-streptomycin (PS; Gibco, Waltham, USA). Cells were incubated at 37°C under a humidified atmosphere of 5% CO_2_. Passaging of cells was performed once the confluence reached 80%. Cells were washed with PBS and TrypLE (Gibco) was applied for cell dissociation. Following incubation with TrypLE for 5 minutes at 37°C, a complete medium was added to the cells. The cell suspension was centrifuged at 500 g for 5 minutes. Seeding densities for ECM Select Array and CytoSoft plate were 0.3×10^5^ cells/mL and 0.75×10^5^ cells/mL, respectively. The samples were then incubated for 24 hours prior to staining. We performed a series of tests on Cytosoft plates to optimise the seeding density for microscopy. The high growth rate of MDA-MB-231 cells resulted in a confluency build-up that can hinder accurate cell segmentation in case of excessive overlapping. This precluded cell culturing over longer periods.

### Staining procedures

ECM Select Array was washed with 5 mL warm 1x Hank’s Balanced Salt Solution with Ca^2+^ and Mg^2+^ (HBSS; Gibco). Cells were then fixed on the slide by adding 5 mL cold 4% paraformaldehyde (PFA) prepared from methanol-free 16% formaldehyde solution (Thermo Fisher Scientific, Waltham, USA) in 1x PBS. The slide was left in the PFA solution for 5 minutes at 4°C followed by 10 minutes at room temperature and then washed again with 1x HBSS. To visualise the cell nuclei, cells were stained with 4,6-diamidino-2-phenylindole dihydrochloride (DAPI, Sigma-Aldrich). The slide was covered with PBS for imaging.

Cells in CytoSoft plates were fixed with 4% PFA and left for 15 minutes at room temperature. Membranes of fixed cells were labelled with FITC-conjugated WGA (Sigma-Aldrich, 1:300) and diluted in HBSS for 10 minutes at 37°C. Next, cells were permeabilised using 0.2% Triton in PBS for 15 minutes at room temperature and then blocked using 1% Bovine Serum Albumin (BSA; Sigma-Aldrich) in PBS for 1 hour at room temperature. Half of the wells on each plate were stained for E-cadherin and the other half for vimentin and cytokeratins (Fig C in S1 Text). Samples were incubated for 1 hour at room temperature with the following primary antibodies: either mouse anti-Vimentin (1:100; Invitrogen, Waltham, USA) and rabbit anti-pan Cytokeratin (1:100; Abcam, Cambridge, UK) or rat anti-E-cadherin (1:200; Sigma-Aldrich). To perform fluorescent microscopy, cells were then treated for 1 hour at room temperature with fluorophore-labelled secondary antibodies against the corresponding host species: goat anti-rabbit Alexa Fluor 647 (1:300; Abcam), goat anti-mouse Alexa Fluor 568 (1:300; Invitrogen), goat anti-rat Alexa Fluor 647 (1:200; Abcam). DAPI was incorporated for the last 10 minutes of incubation followed by washing with PBS. All primary and secondary antibodies were diluted in blocking solution. Finally, 1 mL of PBS was added into each well and plates were stored at 4°C prior to imaging. Our measurements show that the stiffness has a negligible effect on the optical properties of the fluorescent labels used in our immunocytochemistry assays (as discussed in S8 Text).

### Image acquisition

To evaluate the cell adherence to different ECM proteins, images were acquired using an upright epi-fluorescent microscope Axio Imager Z2 (Zeiss, Germany) with a dry EC Plan-Neofluar 5×/0.16 objective. To study the effect of substrate stiffness, high magnification imaging was carried out using an inverted confocal laser-scanning microscope (LSM 880, Carl Zeiss, Germany) equipped with an oil-immersion Plan-Apochromat 40×/1.3 objective lens. The cell nuclei stained with DAPI were imaged under excitation with a 405 nm laser and the emission filter was tuned to 411–494 nm. WGA-FITC was excited with a 488 nm laser and emission collected through a 491–544 nm bandpass filter. Vimentin was excited with a 561 nm laser and the emission bandpass filter was in the 568–620 nm range. All images for pan cytokeratin and E-cadherin were acquired under excitation with a 633 nm laser and emission was detected over the 638–755 nm range. To avoid signal overlapping, imaging was performed sequentially for each fluorophore with frame-wise switching between channels. 9–15 fields of view were randomly selected in each well for image acquisition. Cell density was calculated from images obtained by the same system with a dry Plan-Apochromat 10×/0.45 objective.

### Image analysis and cell segmentation

Acquired images containing signal from cell membranes and nuclei were preprocessed and segmented in ImageJ v2.0.0, [50]. Noise reduction was performed with a Gaussian filter with kernel σ = 0.25um. Colour thresholding was applied to separate cells and nuclei from the background followed by manual segmentation of adjacent cells to ensure the highest precision. Cells not completely present in the image or overlapping with other cells were excluded from the further single-cell analysis. However, they were taken into account when calculating context measurements such as the number of cell neighbours or the length of the shared boundary. Created images with masked cells and nuclei were used to extract features from individual cells. The complete dataset contained 910 cells. Table A in S2 Text provides the exact number of segmented cells per condition.

### Feature extraction

Automated feature extraction was performed using CellProfiler 3.1.8 software [51]. Measurements of cell and nuclei shape, context measurements, intensity and texture of DNA, membrane, cytokeratins and vimentin were obtained using built-in plugins in CellProfiler. First, variables with zero variance and absolute pixel coordinates were removed from the dataset. Additional features were engineered, such as measurements of cell polarity (distance between centroids of a cell and its nucleus), nuclear-cytoplasmic ratio (ratio of the areas of a nucleus and a cytoplasm) and the fraction of fluorescent signal localised on edge. Specifically, throughout the paper, we refer to the total fluorophore intensity normalised by cell cross-sectional area as mean intensity. Similarly, mean edge intensity denotes fluorophore intensity captured from the boundary pixels normalised by cell perimeter. The fraction of the total cell fluorescent signal captured along the edge of a cell was calculated as the total edge intensity / total cell intensity * 100.

Overall, 150 measurements were calculated for each cell describing its morphology and context (Table B in S2 Text). Additionally, depending on the staining group, 140 measurements were obtained describing the intensity and distribution of fluorescent signal from cytokeratins and vimentin and 69 measurements describing the expression of E-cadherin.

### Hierarchical clustering

All features were standardised prior to further analysis by subtracting the mean from each column and dividing it by its standard deviation (z-score normalisation). That allowed to take all features into account with equal weights regardless of the scale and the nature of the variable.

Cluster validity indices were used to estimate the number of clusters in the dataset. This procedure was performed by randomly subsampling ~30% of the data and was repeated 30 times. Two alternative indices, namely the silhouette score [52] and the Davies-Bouldin index [53], supported the presence of 2 to 3 clusters in the dataset. Taking into account the cluster validity indices and examining the outcomes of the hierarchical clustering we concluded that cells exhibit 3 general morphotypes.

Agglomerative hierarchical clustering was performed on the whole population of cells using all 150 morphological and contextual features recursively merging clusters subject to the smallest variance. The output was displayed as a dendrogram.

### Feature selection

We performed iterative feature selection removing features with Pearson’s correlation coefficient > 0.8. As a result, 50 features were excluded, and the rest 100 features were used to assess cluster separability and train a Random Forest model.

### Statistical analysis

For the cell adhesion study, we computed Spearman’s rank correlation coefficient to determine the associations between protein concentrations and the number of attached cells. To identify cell properties associated with the substrate stiffness value, we calculated and compared the results of Spearman’s and Kendall’s rank correlation coefficients as well as distance correlation [54] that can capture both monotonic and non-monotonic relationships. For feature selection, we only considered Pearson correlation. Features correlated with cluster number and cell clumping were identified using the mutual information criterion. Bar plots show mean and error bars indicate standard deviation unless otherwise stated. Distributions of the values are shown using box plots, where a box represents the median value, first and third quartiles, whiskers indicate median +/-1.5 * IQR. Confidence intervals were calculated at the 95% level both for mean values and proportions.

Statistical discernibility was assessed using unpaired two-tailed Student’s t-test with Welch’s correction for unequal variances. Statistical significance was reported as follows: *p-value < 0.05, **p-value < 0.01 and ***p-value < 0.001.

All experiments were replicated three times and for each sample, nine to twelve sections were randomly selected and captured by confocal microscope.

### Random Forest classification model

A Random Forest Classifier was used to predict the cell morph based on the selected 100 morphological and contextual features. The dataset was split into training and test set with 10% of the data used for model evaluation. Hyperparameter tuning was performed using randomised search with K-fold cross-validation to optimise the model parameters and achieve the best results. The performance of the model was evaluated using the F_1_ score (a weighted average of the precision and recall) due to unequal sizes of the classes.

### In silico analysis

Updated clinical data and sample genomic information for TCGA-BRCA samples were obtained from the Genomic Data Commons (https://portal.gdc.cancer.gov/). All data analysis including gene expression, mutation, copy number variation (CNV), and Pearson´s correlation coefficient was performed with TCGAbiolinks and Bioconductor packages under R program (version 3.4.0). The ECM-signature (18 genes) and classical EMT-signature (14 genes) used in this analysis were analysed and selected according to the previous studies [38,55,56].

## Supporting information

S1 Text Experimental details. (DOCX)

S2 Text Multi-parametric image-based cell profiling. (DOCX)

S3 Text Exploratory data analysis of single-cell profiles. (DOCX)

S4 Text Hierarchical clustering. (DOCX)

S5 Text Expression of biomarkers. (DOCX)

S6 Text Analysis of multicellular clusters at 64 kPa. (DOCX)

S7 Text Correlation between ECM stiffness and EMT signature. (DOCX)

S8 Text Fluorescent quantum yield vs microenvironment stiffness. (DOCX)
