# Supplementary material for: Machine learning reveals mesenchymal breast carcinoma cell adaptation in response to matrix stiffness
Source: PLoS Comput Biol. 2021 Jul 23;17(7):e1009193. doi: 10.1371/journal.pcbi.1009193 (PMC8336795; doi:10.1371/journal.pcbi.1009193)
Supplement: S1 Text — (DOCX) [file pcbi.1009193.s001.docx]

Experimental details

MDA-MB-231 adhesion to different ECM proteins

Cells display varying adherence to different components of the extracellular matrix (ECM). To reveal which ECM proteins are the most attractive for triple-negative breast cancer cells, we used a commercial extracellular matrix screening array, ECM Select Array Kit Ultra-36 (Advanced Biomatrix). We tested 36 combinations of nine ECM proteins taken in different concentrations (Table A). Each condition was replicated in 9 spots imprinted on hydrogel surface and the non-fouling material of the rest of the slide ensured efficient cellular attachment (Fig A). Cells were cultured for 24 hours, fixed, washed and labelled with DAPI. Cell numbers were evaluated by counting the number of nuclei located within each spot. The number of adherent cells in case of a single protein coating are shown in Table B. Fig B shows the results for all ECM protein combinations demonstrating that quite a few different conditions provide high adhesion of MDA-MB-231 cells. Next, we performed correlation analysis to determine which ECM protein has the strongest association between its concentration and the number of attached MDA-MB-231 cells.

The list of ECM proteins tested for TNBC cell adhesion:

1. Collagen, Human, Type I
2. Collagen, Human, Type III
3. Collagen, Human, Type IV
4. Collagen, Human, Type V
5. Collagen, Human, Type VI
6. Fibronectin, Human
7. Laminin, Human
8. Vitronectin, Human
9. Tropoelastin, Human, Recombinant

**
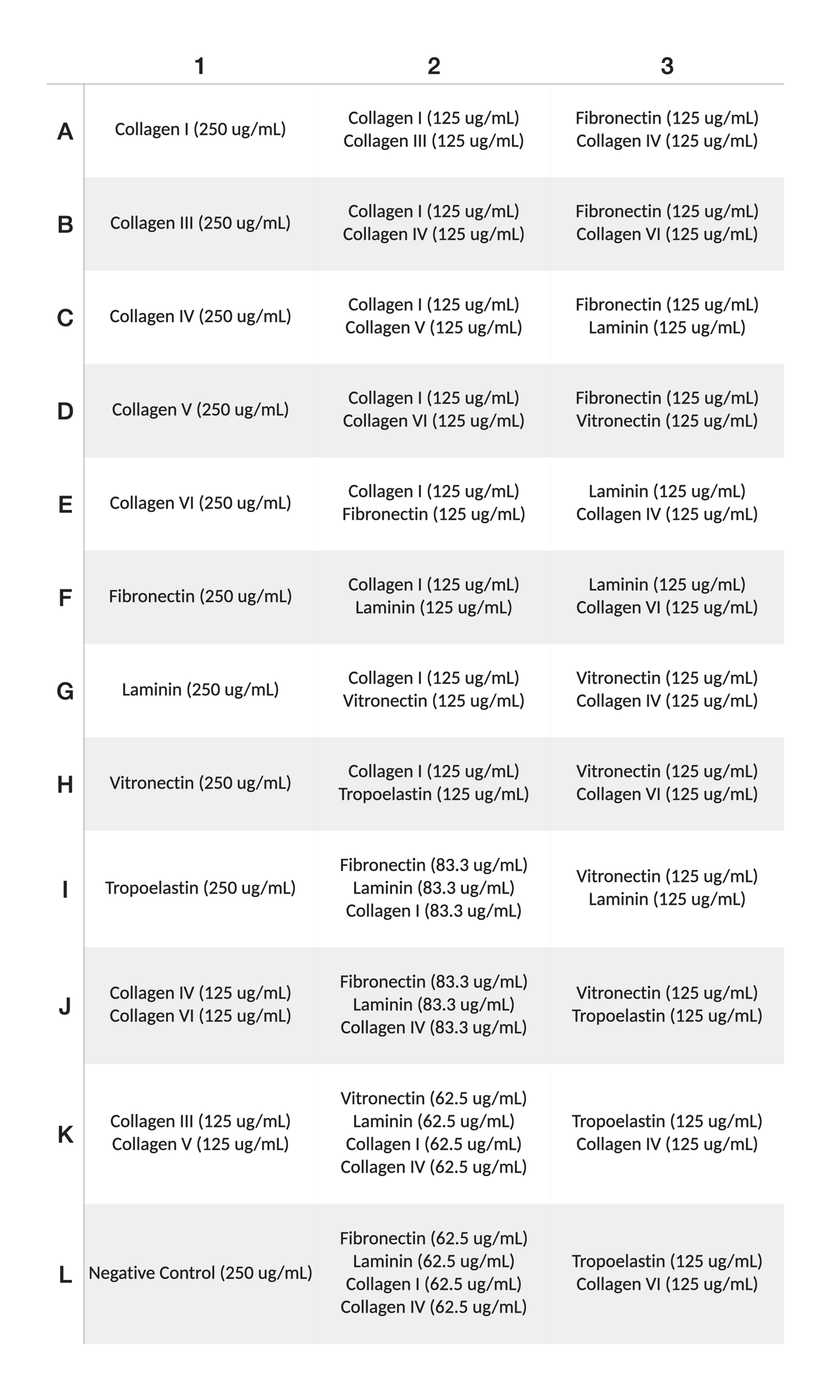
**

**Table A**. The arrangement of the ECM conditions on the microarray used to evaluate TNBC cell adhesion to different ECM proteins. Negative control was located in the cell L1 of the array.


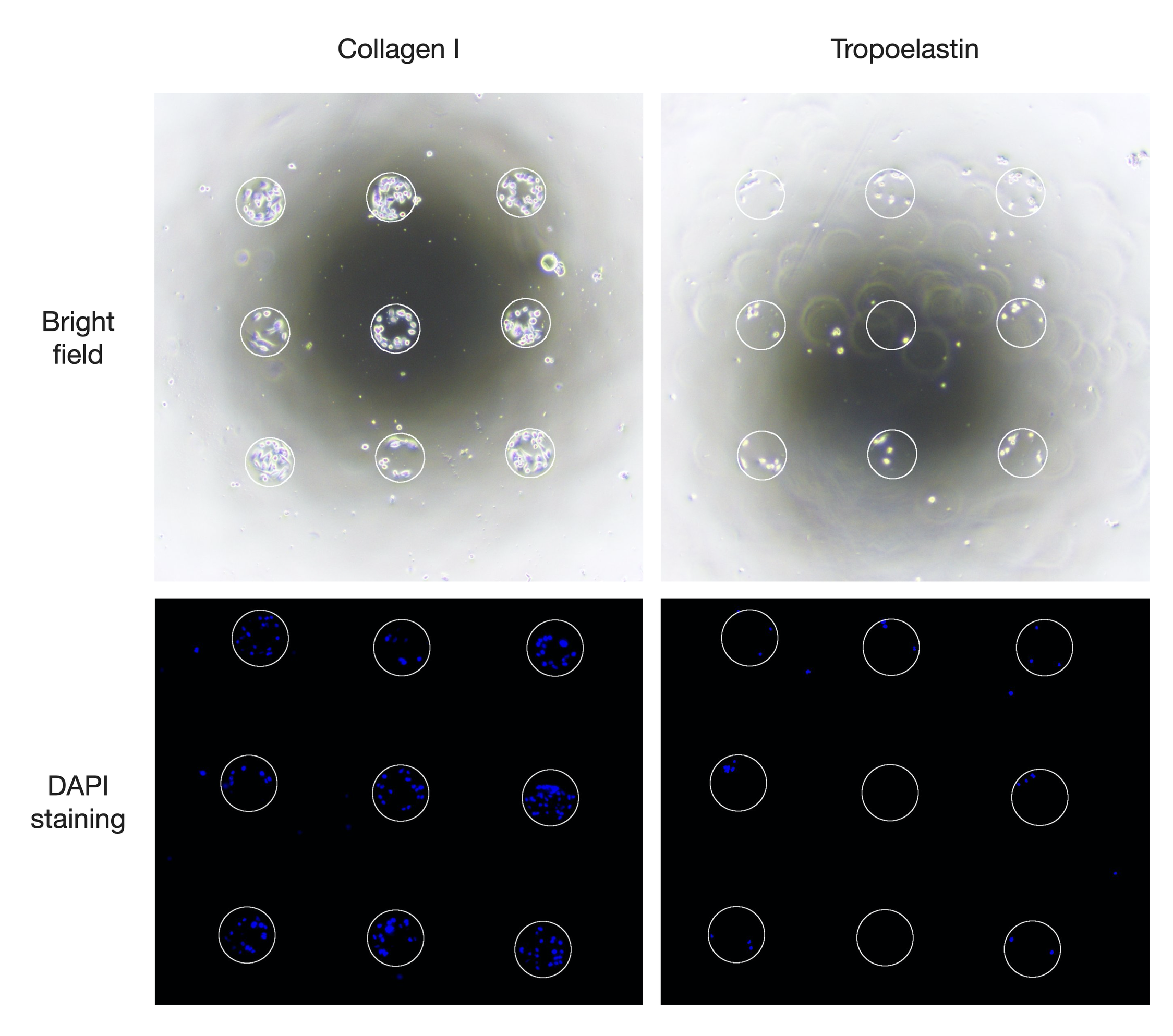


**Fig A.** Raw images of cells showing high adherence (collagen 1) and low adherence (tropoelastin): bright-field images (top), DAPI staining (bottom). Note that bright-field images were acquired before washing, hence, may show more cells. Cell numbers were calculated from the DAPI stained images by performing nuclei segmentation (see Materials and Methods).


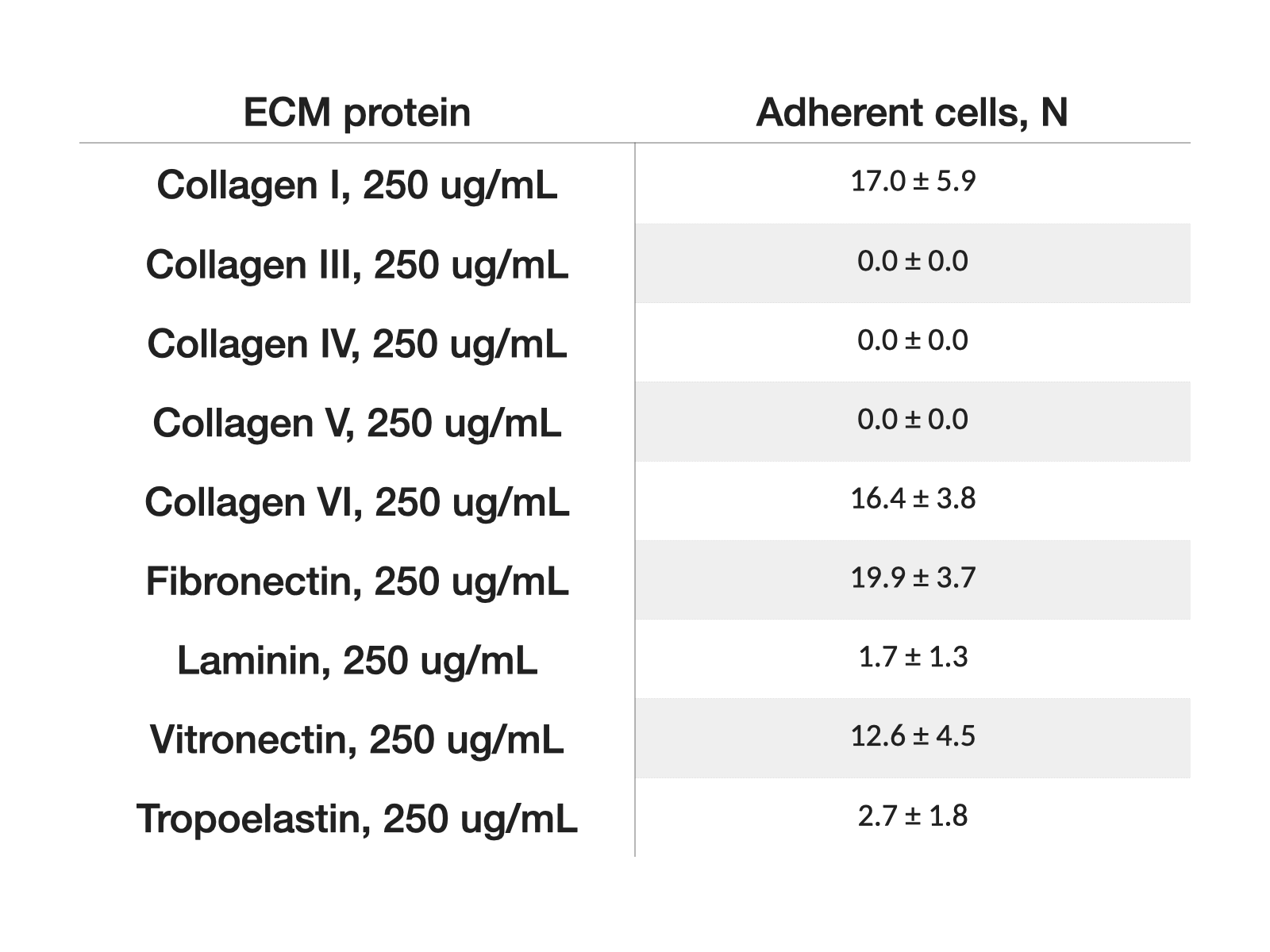


**Table B**: The number of adherent cells in case of a single protein coating. Values are mean ± standard deviation, n = 9 replicates.


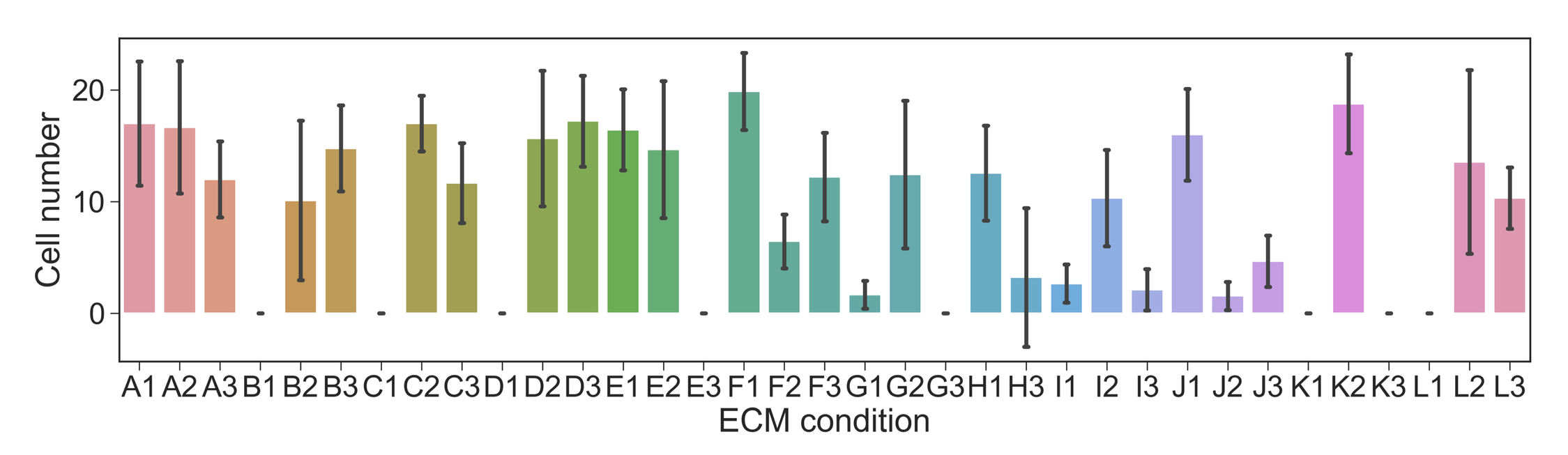


**Fig B.** The number of cells attached to spots coated with different ECM conditions. Each condition is denoted by a letter and a digit as per the Table A. Bars indicate mean ± standard deviation. Each condition was replicated 9 times.

## Staining procedures

MDA-MB-231 were seeded on commercial CytoSoft Imaging 24-well plates (Advanced Biomatrix) with different substrate stiffness. Cells were cultured for 24 hours and fixed (see Materials and Methods). To investigate changes in the molecular states of the cells, each plate was divided in two parts; half of the wells on each plate were stained for E-cadherin and the other half for vimentin and cytokeratins (Fig C).


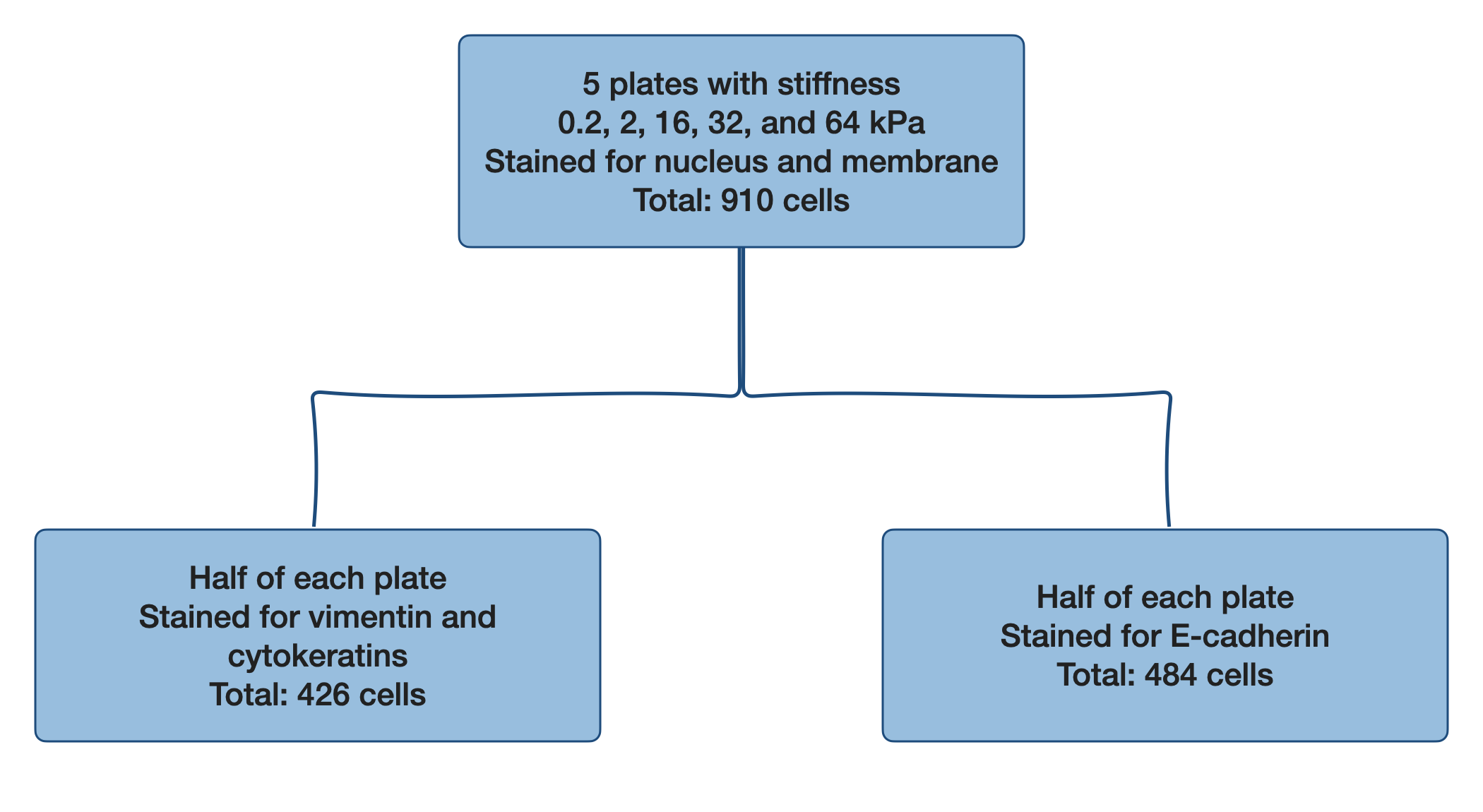


**Fig C.** Diagram indicating how wells with cells were split during the staining procedure to limit the number of fluorophores to 4 and avoid overlapping signals.
