# Supplementary material for: Machine learning reveals mesenchymal breast carcinoma cell adaptation in response to matrix stiffness
Source: PLoS Comput Biol. 2021 Jul 23;17(7):e1009193. doi: 10.1371/journal.pcbi.1009193 (PMC8336795; doi:10.1371/journal.pcbi.1009193)
Supplement: S2 Text — (DOCX) [file pcbi.1009193.s002.docx]

# Multi-parametric image-based cell profiling


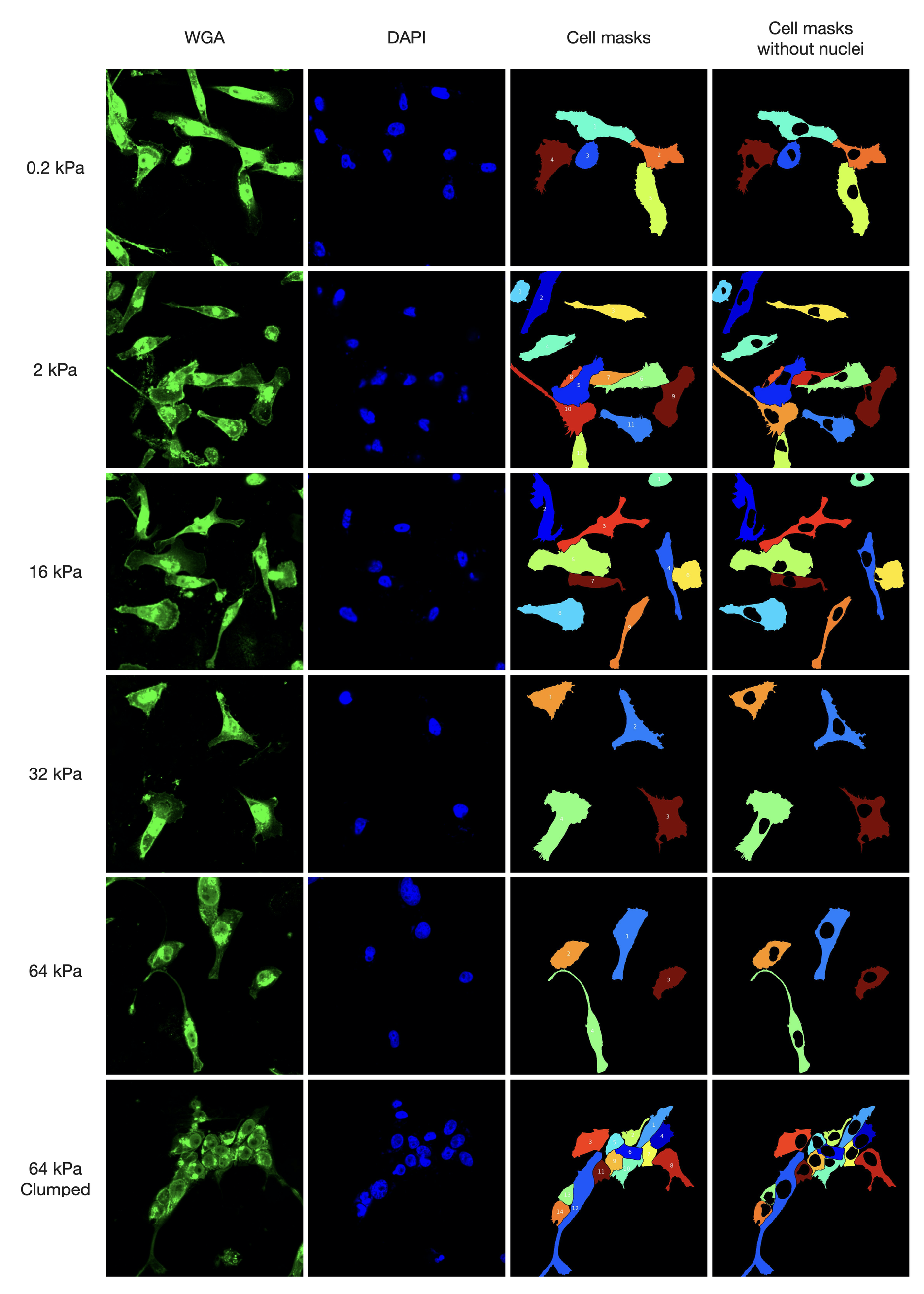


**Fig A.** Representative images of cell segmentation at each stiffness level plus segmentation of multicellular clusters found at 64 kPa. Cell membranes and nuclei were visualised with WGA and DAPI staining. The extracted cell masks with and without nuclei are presented in the right two columns.


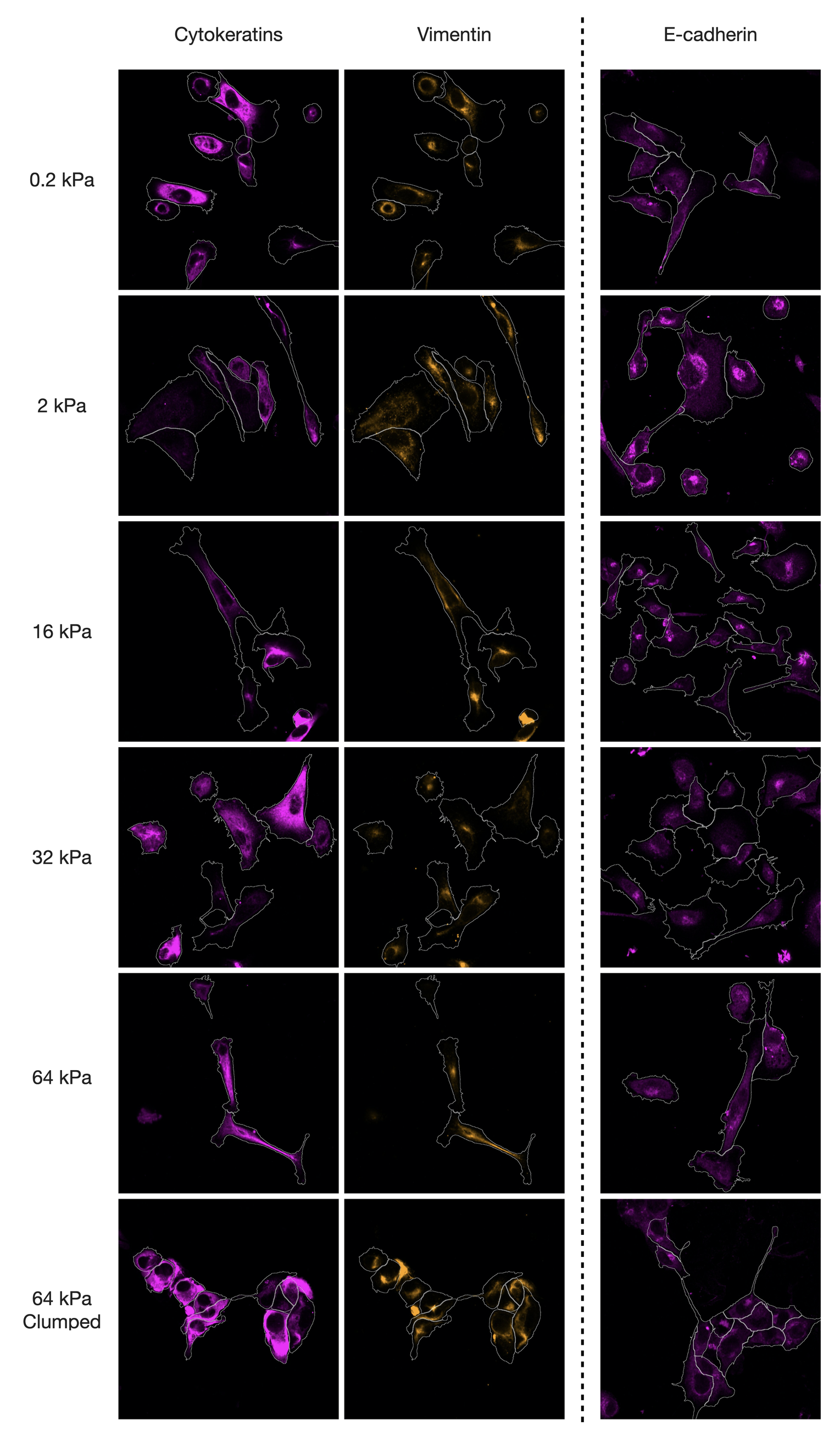


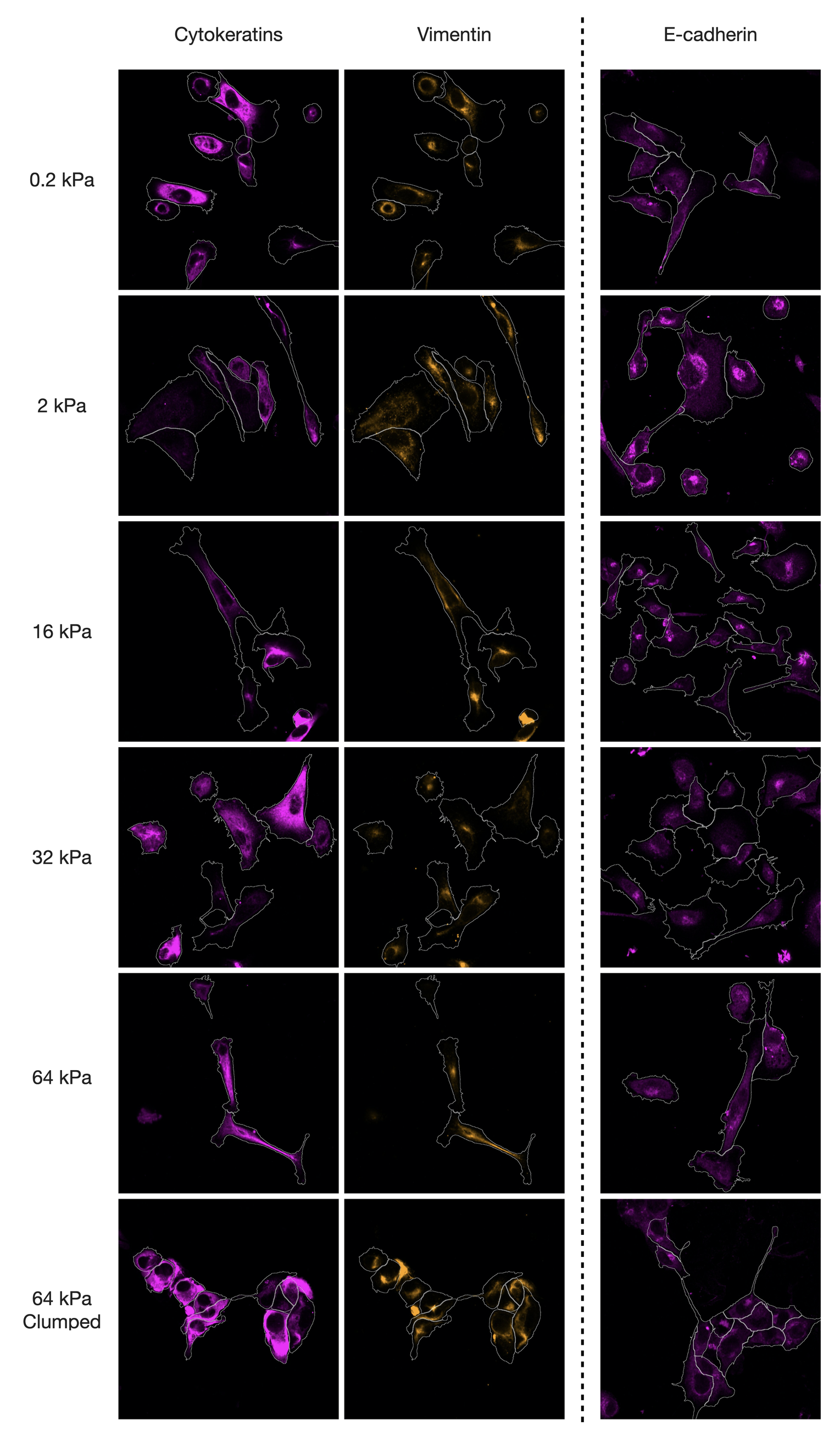
**Fig B.** Representative images of cells stained for cytokeratins and vimentin (left and middle columns) and E-cadherin (right column) at each stiffness level plus staining of multicellular clusters found at 64 kPa.


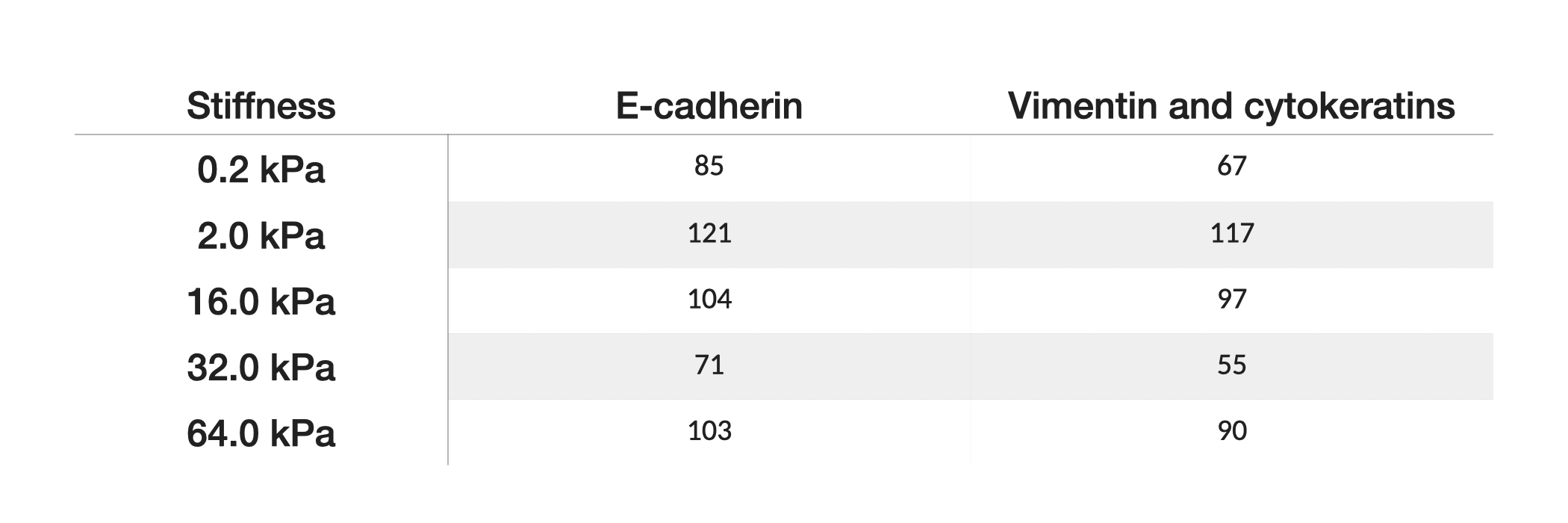


**Table A.** The total number of segmented cells per stiffness value stained either for E-cadherin or vimentin and cytokeratin.


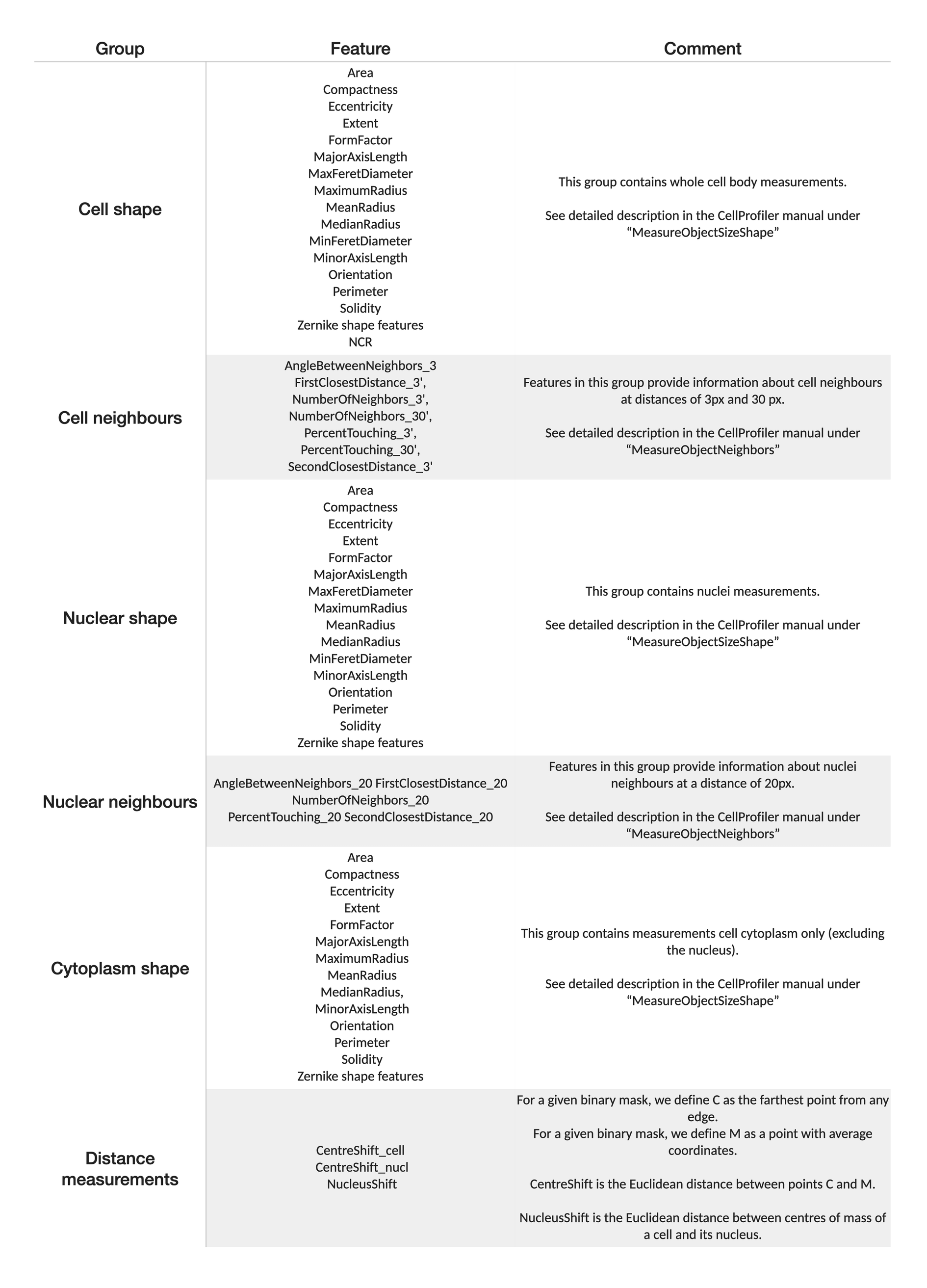


**
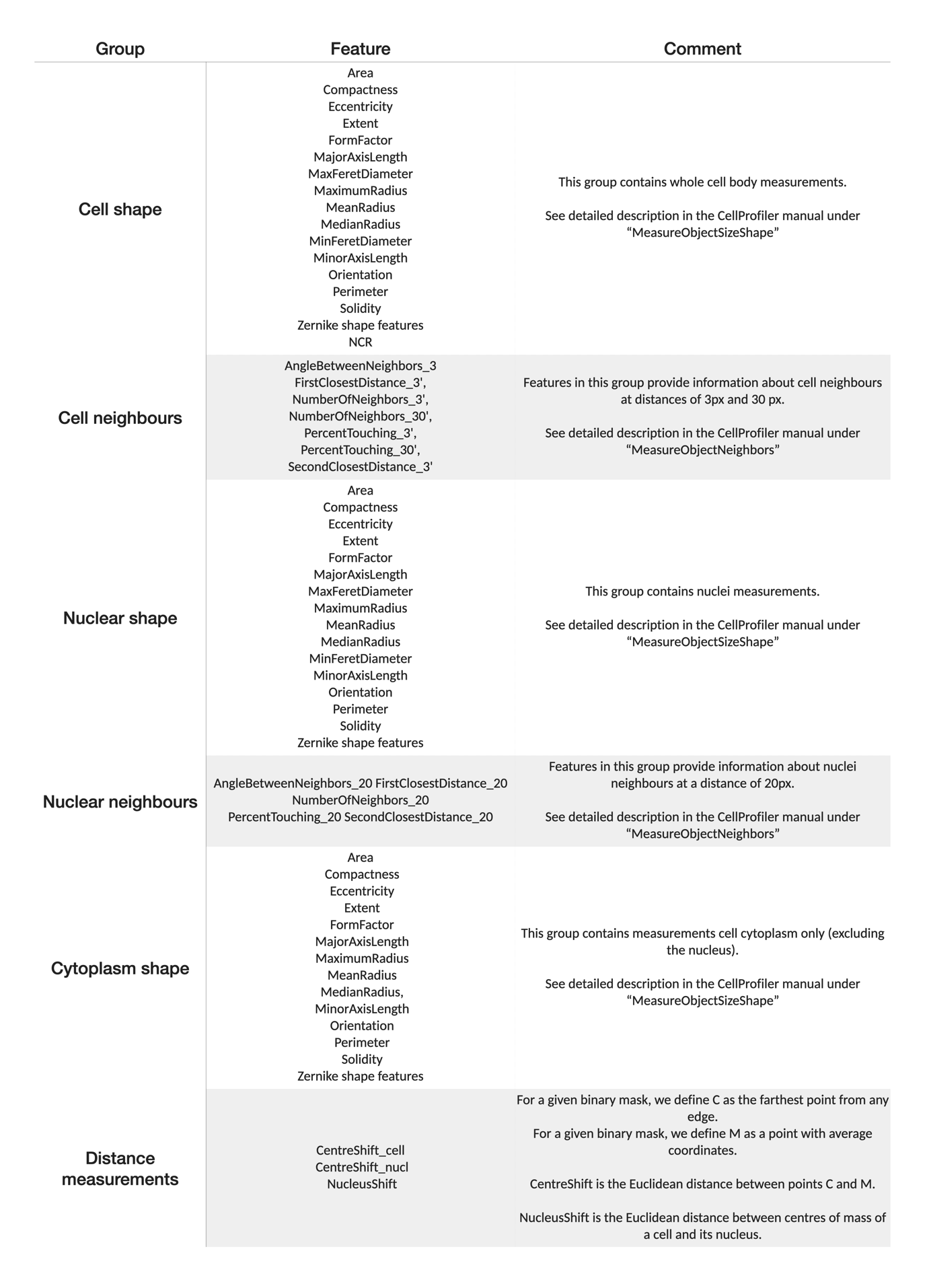
**

**Table B.** The full list of morphological and contextual measurements extracted to generate single-cell profiles. Link to the CellProfiler manual: <http://cellprofiler-manual.s3.amazonaws.com/CellProfiler-3.0.0/index.html>


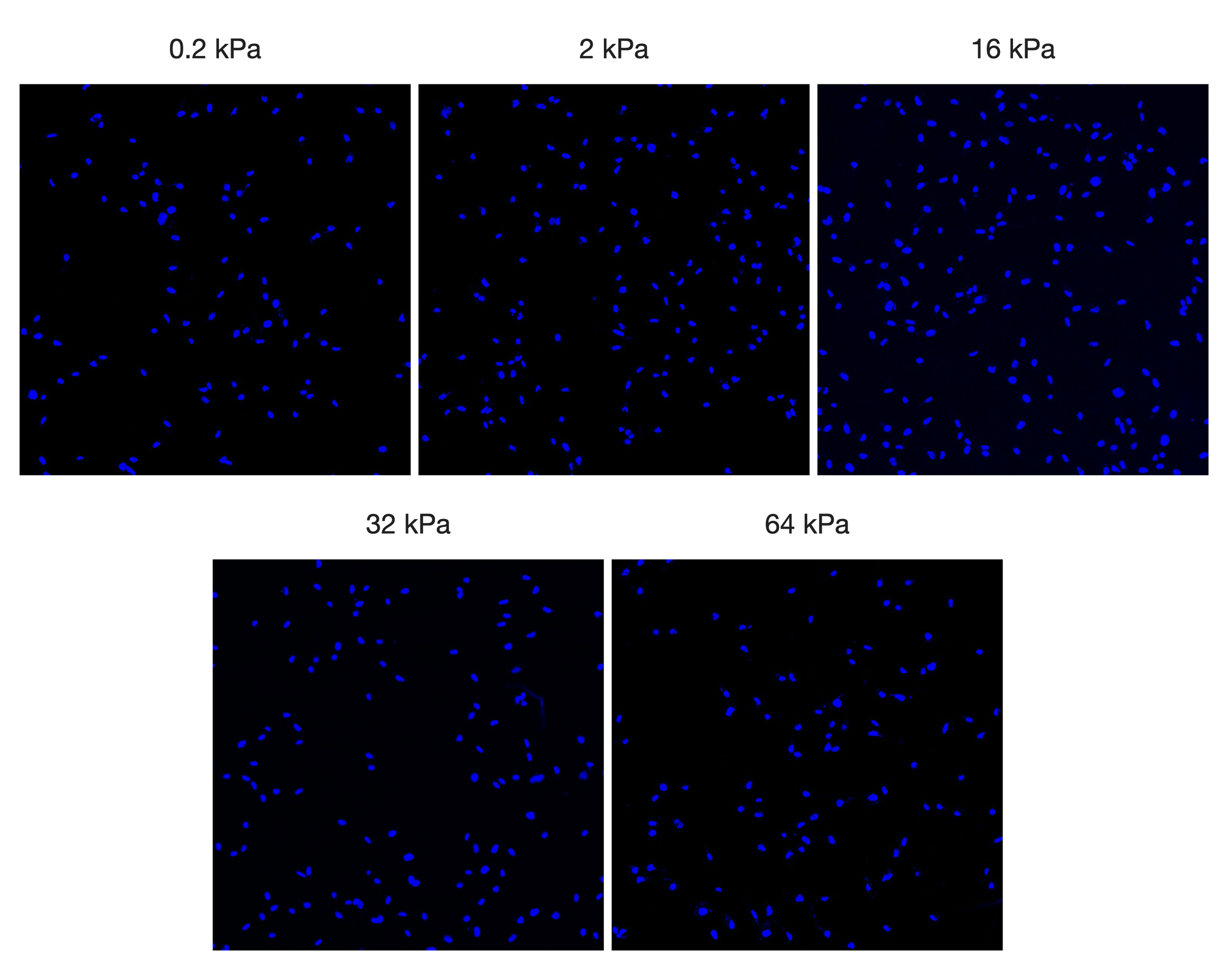


**Fig C.** Representative images taken at low magnification for cell density evaluation at different stiffness levels.
