# Supplementary material for: Machine learning reveals mesenchymal breast carcinoma cell adaptation in response to matrix stiffness
Source: PLoS Comput Biol. 2021 Jul 23;17(7):e1009193. doi: 10.1371/journal.pcbi.1009193 (PMC8336795; doi:10.1371/journal.pcbi.1009193)
Supplement: S3 Text — (DOCX) [file pcbi.1009193.s003.docx]

# Exploratory data analysis of single-cell profiles

As described in the main text, single-cell profiles were generated for a total of 910 cells. Among them, 84 cells cultured on the most rigid substrate (64 kPa) formed multicellular clusters and were examined separately. The results shown below were obtained using the main dataset which consisted of the remaining 826 cells, unless stated otherwise.


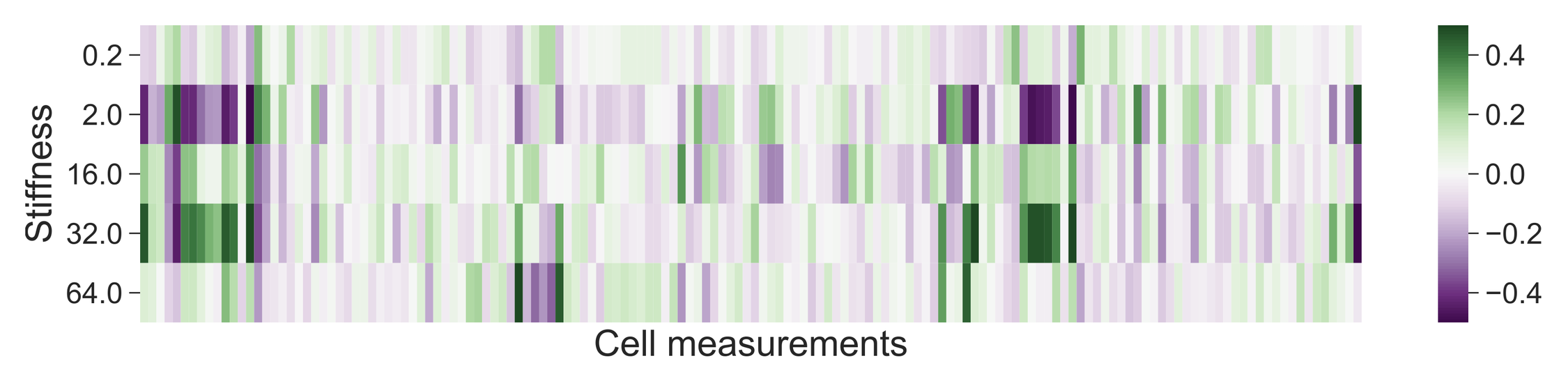
**Fig A.** Individual cells were grouped by substrate stiffness value and average pooling was performed on each of 150 morphological and contextual measurements. Each column corresponds to an individual feature.


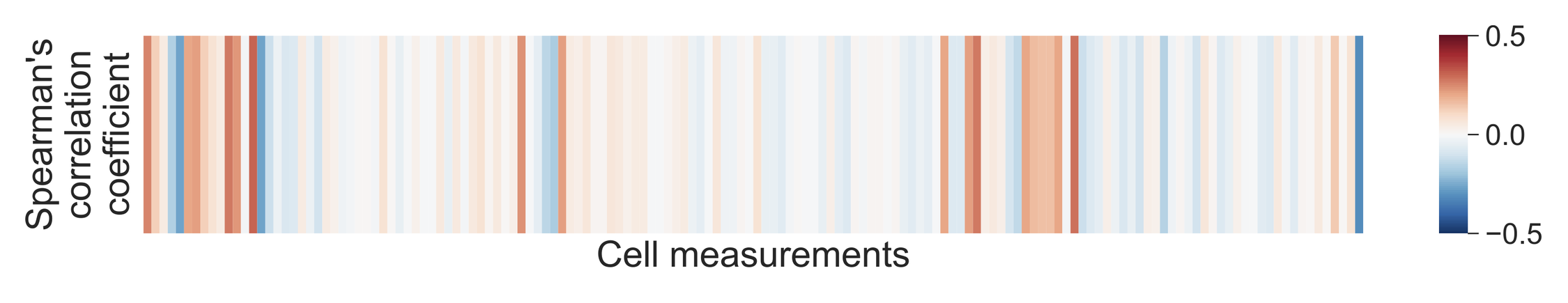
**Fig B.** Correlation between stiffness and different morphological and contextual parameters evaluated by calculating Spearman’s rank correlation coefficient. Each column corresponds to an individual feature. The correlation coefficient assesses if the relationship between two variables can be described using a monotonous function. By definition the coefficient ranges from -1 (blue) to 1 (red), indicating the presence of strong positive and negative correlation, respectively.


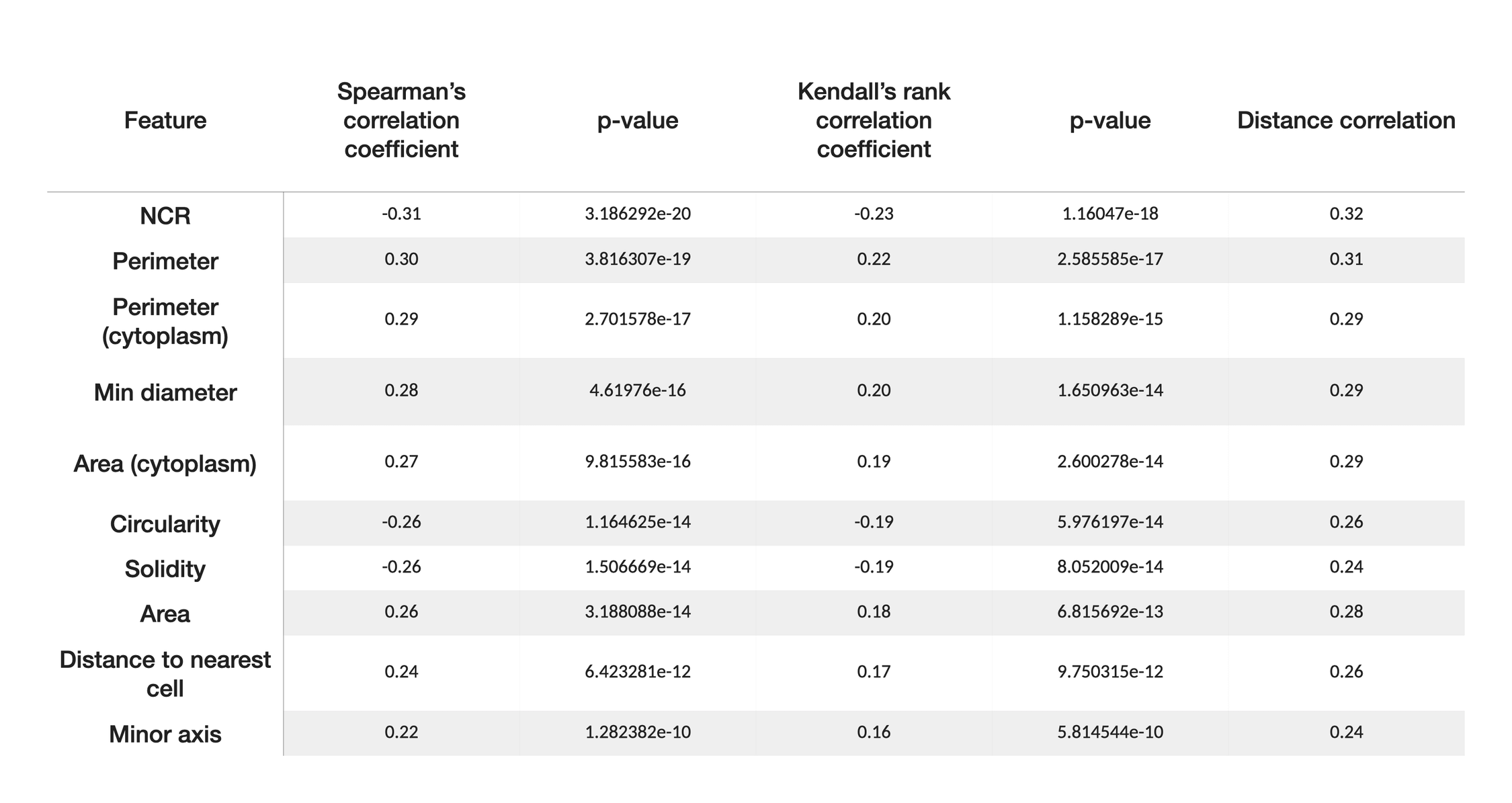


**Table A.** Spearman’s, Kendall’s and distance correlation coefficients identified the same top 10 features that showed the strongest correlation with substrate stiffness.


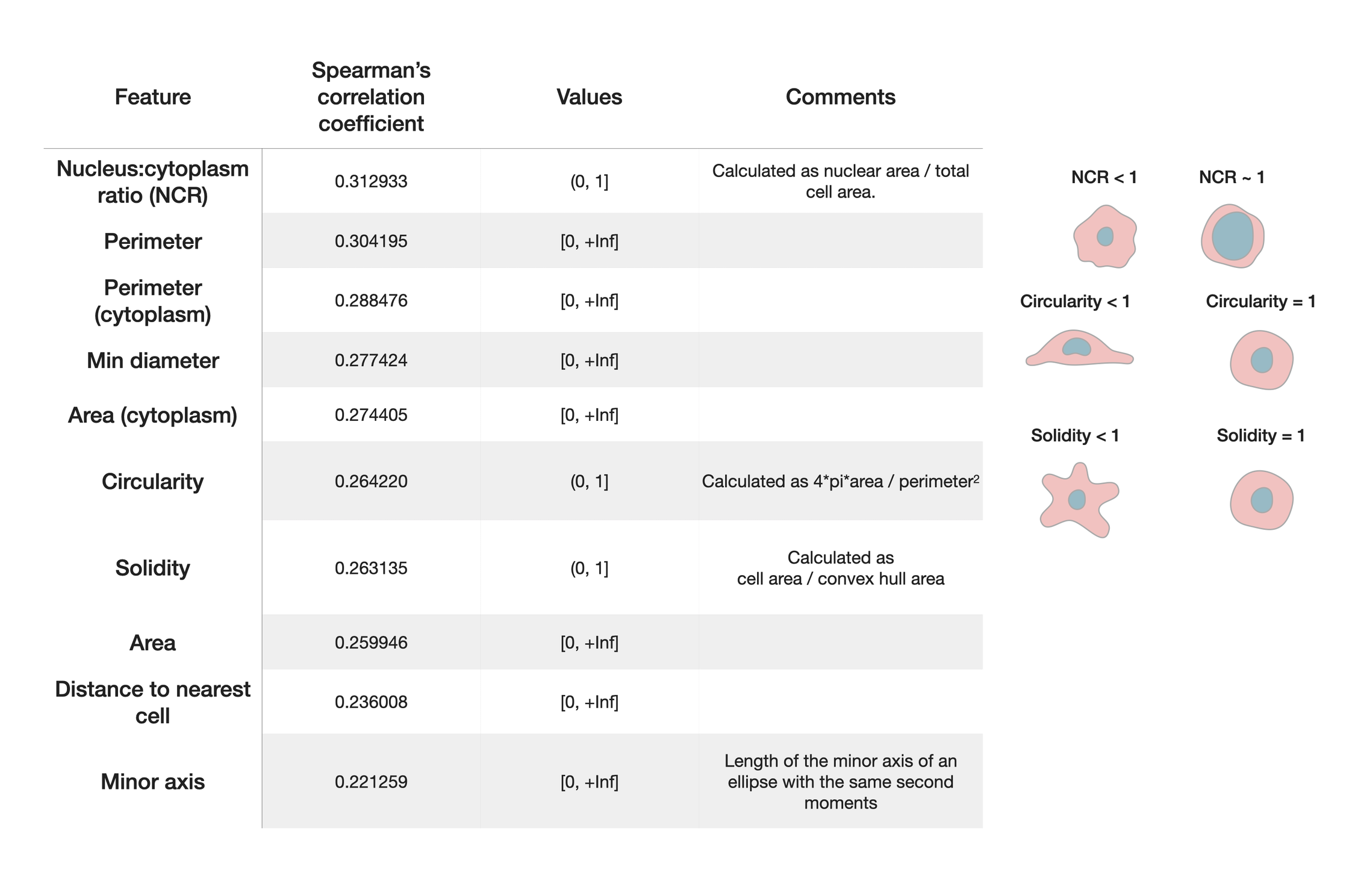


**Table B**. Top 10 features that showed the strongest correlation with substrate stiffness. The table provides the value of Spearman’s correlation coefficient for each feature as well as the range of possible values and some comments on the calculations and illustrations of how the values should be interpreted.


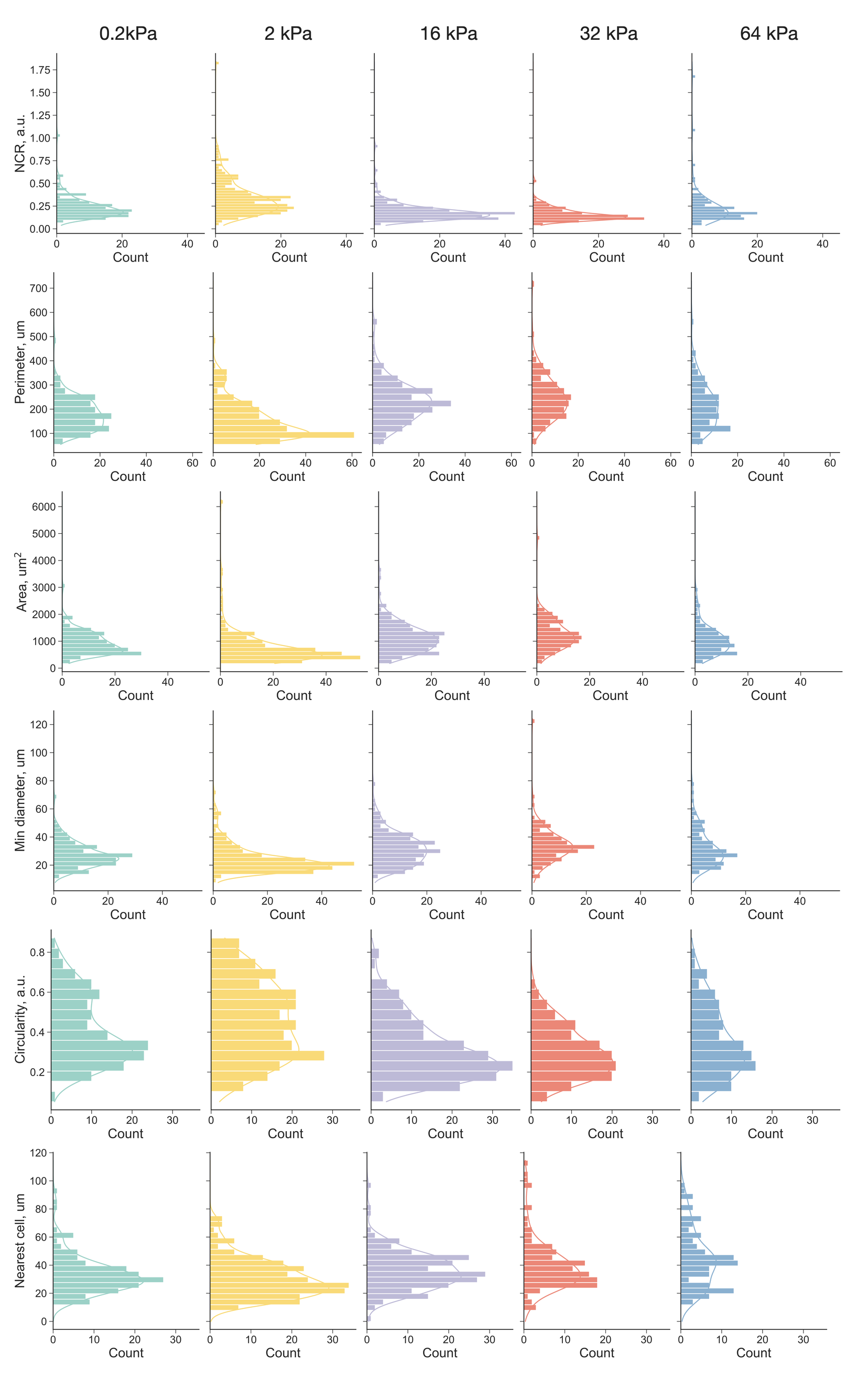


**Fig C.** Histograms show changes in the distributions of NCR, cell perimeter, area, smallest diameter, circularity, and distance to the nearest cell in response to substrate stiffness.
