# Supplementary material for: Machine learning reveals mesenchymal breast carcinoma cell adaptation in response to matrix stiffness
Source: PLoS Comput Biol. 2021 Jul 23;17(7):e1009193. doi: 10.1371/journal.pcbi.1009193 (PMC8336795; doi:10.1371/journal.pcbi.1009193)
Supplement: S4 Text — (DOCX) [file pcbi.1009193.s004.docx]

# Hierarchical clustering

**
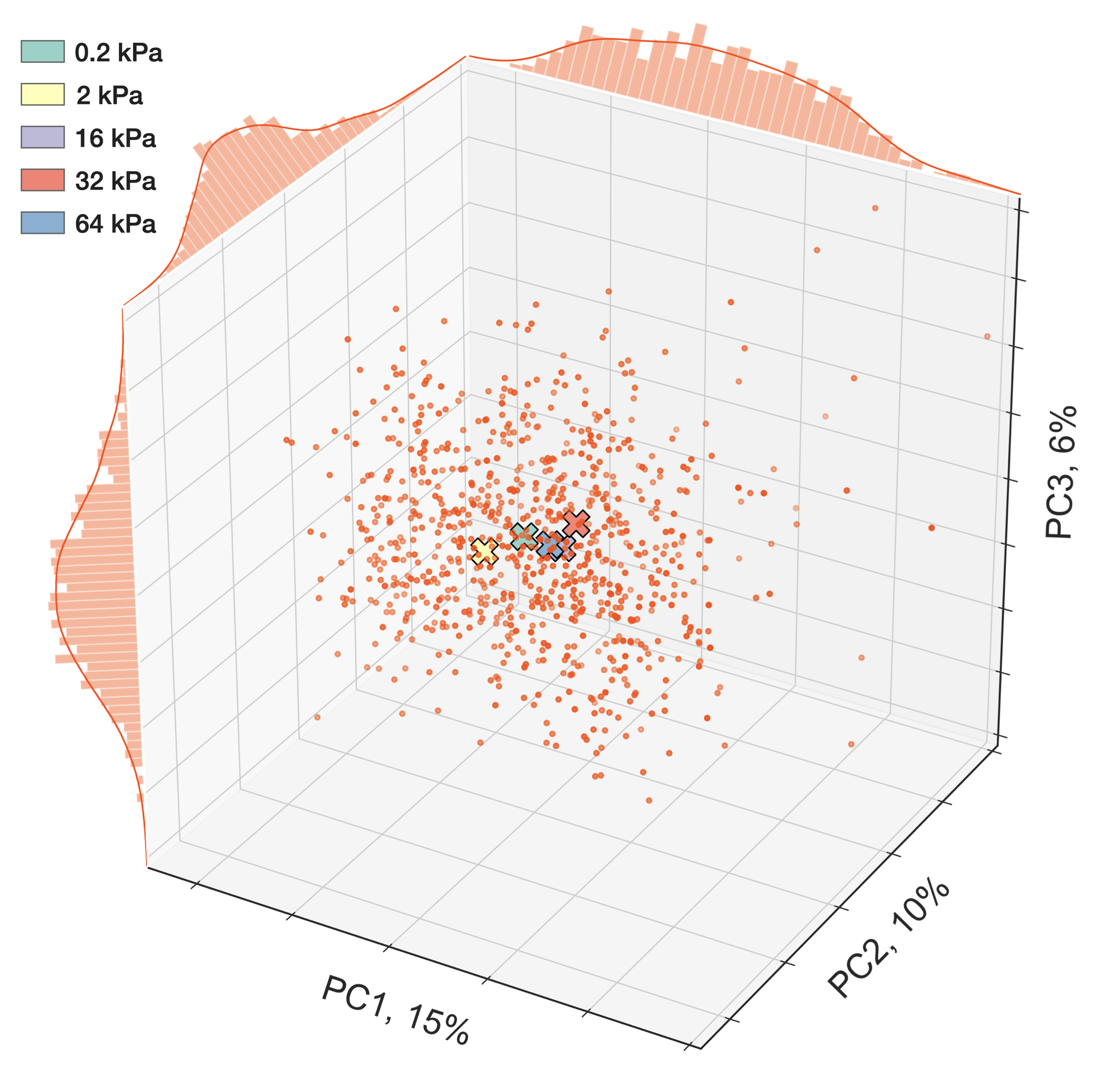
**

**Fig A.** The result of the principal component analysis (PCA) showing the projection of the data on the first 3 principal components (PCs). There appear to be no distinct clusters as confirmed by visualising the histograms of values of each PC. Also, the first 3 PCs account for only 28% of variance suggesting that the data points (i.e., individual cells) are highly variable. “X” markers indicate the centroids of the 5 stiffness groups, e.g., green “X” is the centroid of the group of cells cultured at 0.2 kPa.

Hierarchical clustering was applied to individual profiles of 826 cells to identify subpopulations of cells. To determine the number of subpopulations, two commonly used indices were calculated: Silhouette score and Davies-Bouldin score.


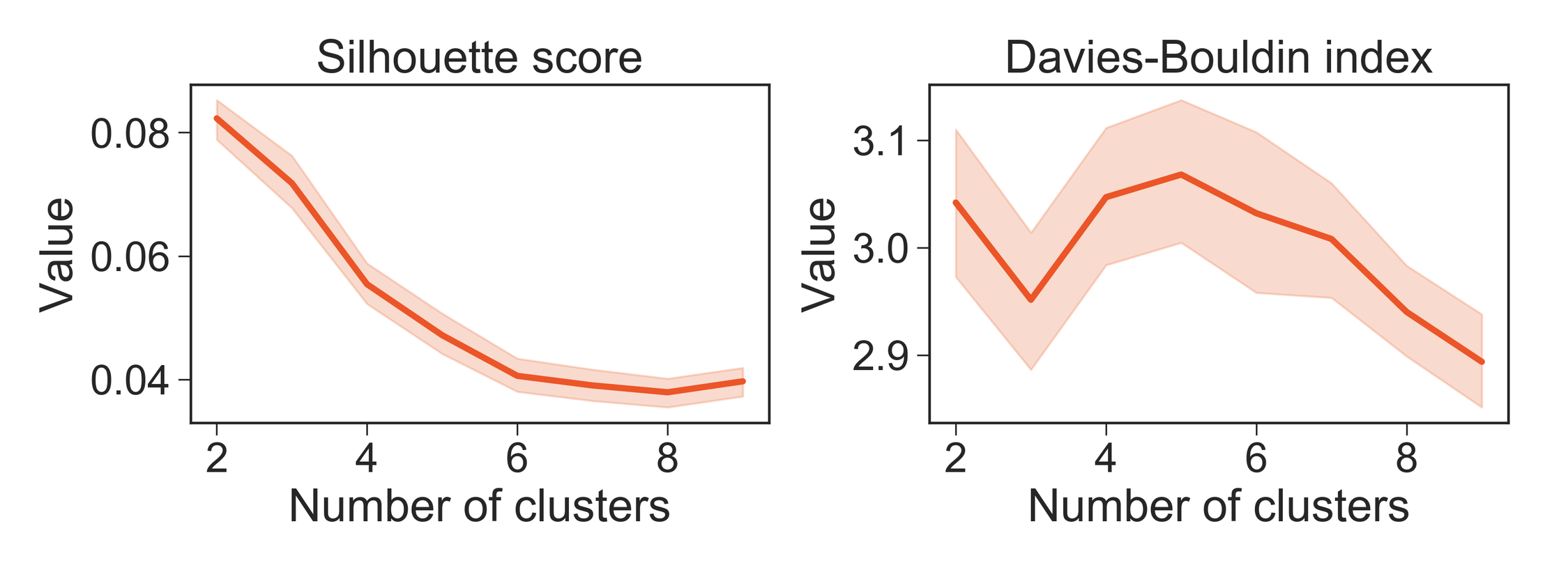


**Fig B.** Cluster validity indices used to estimate the number of clusters to perform hierarchical clustering. The silhouette score ranges from −1 to +1, where a high value indicates that the object is well matched to its cluster and poorly matched to neighbouring clusters. Values near 0 indicate overlapping clusters. For the Davies-Bouldin index, the minimum value is zero, with lower values indicating clusters that are farther apart and less dispersed. Solid lines show the average score, the band indicates 95% CI (see Materials and Methods for details).

Using the output produced by hierarchical clustering, we calculated mutual information to identify features that best delineate the established cell morphs.


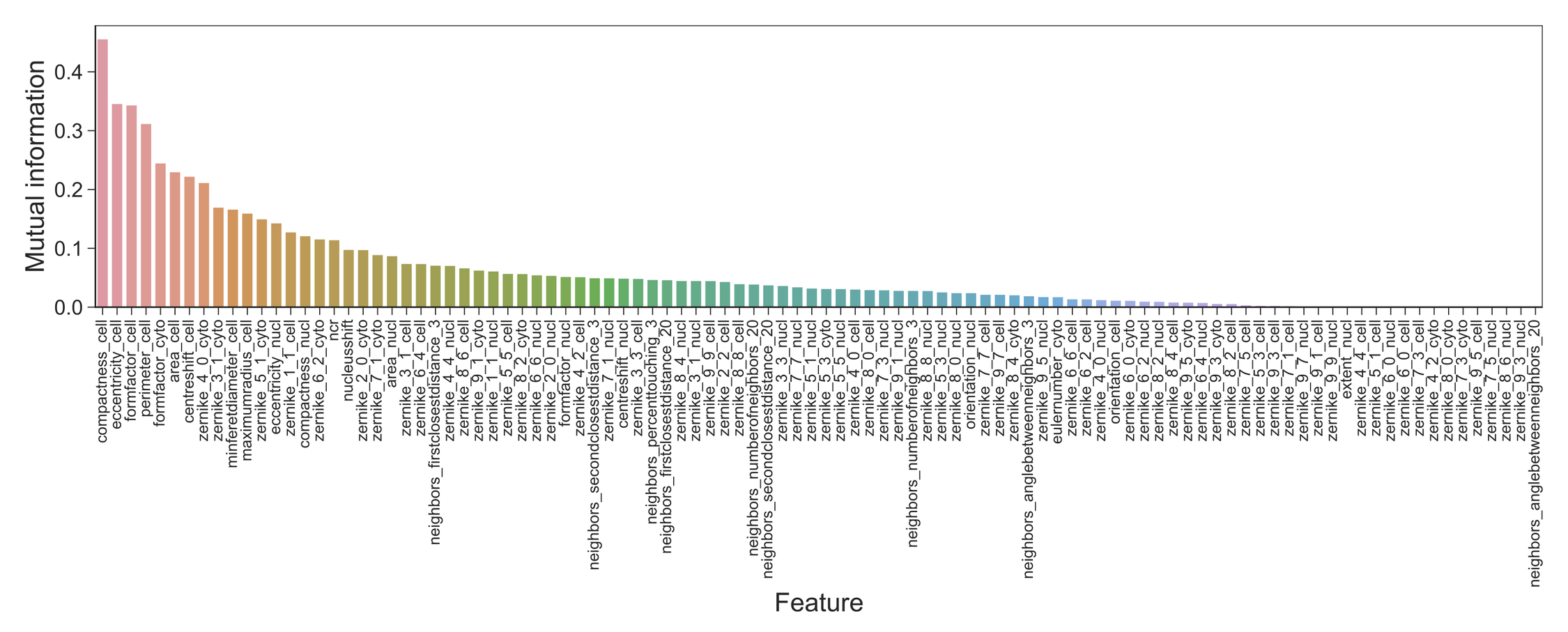


**Fig C**. Contribution of individual morphological and contextual features towards cluster separability as measured the mutual information criterion. Values close to 0 indicates no relationship with the cluster number.

**
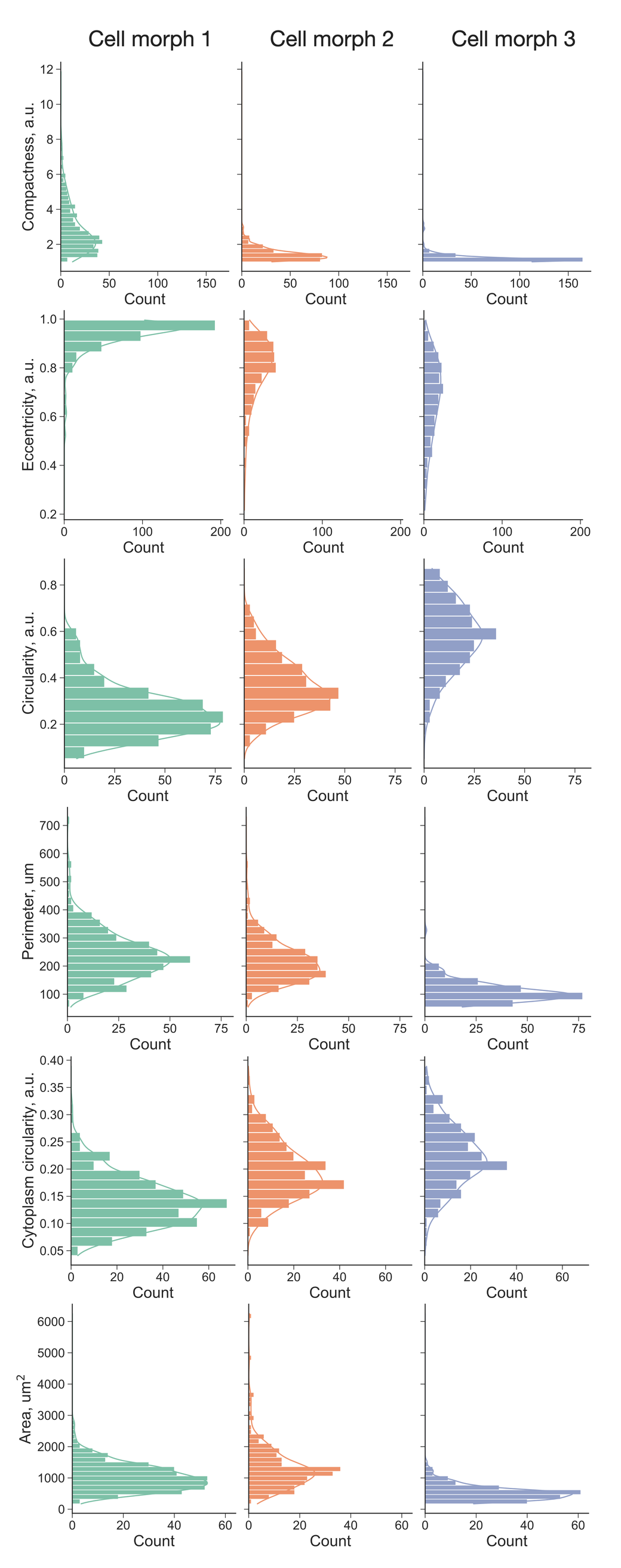
**

**Fig D.** Histograms illustrate the distributions of cell compactness, eccentricity, circularity, perimeter, cytoplasm circularity, and area in subpopulations of cells corresponding to the identified cell morphs.


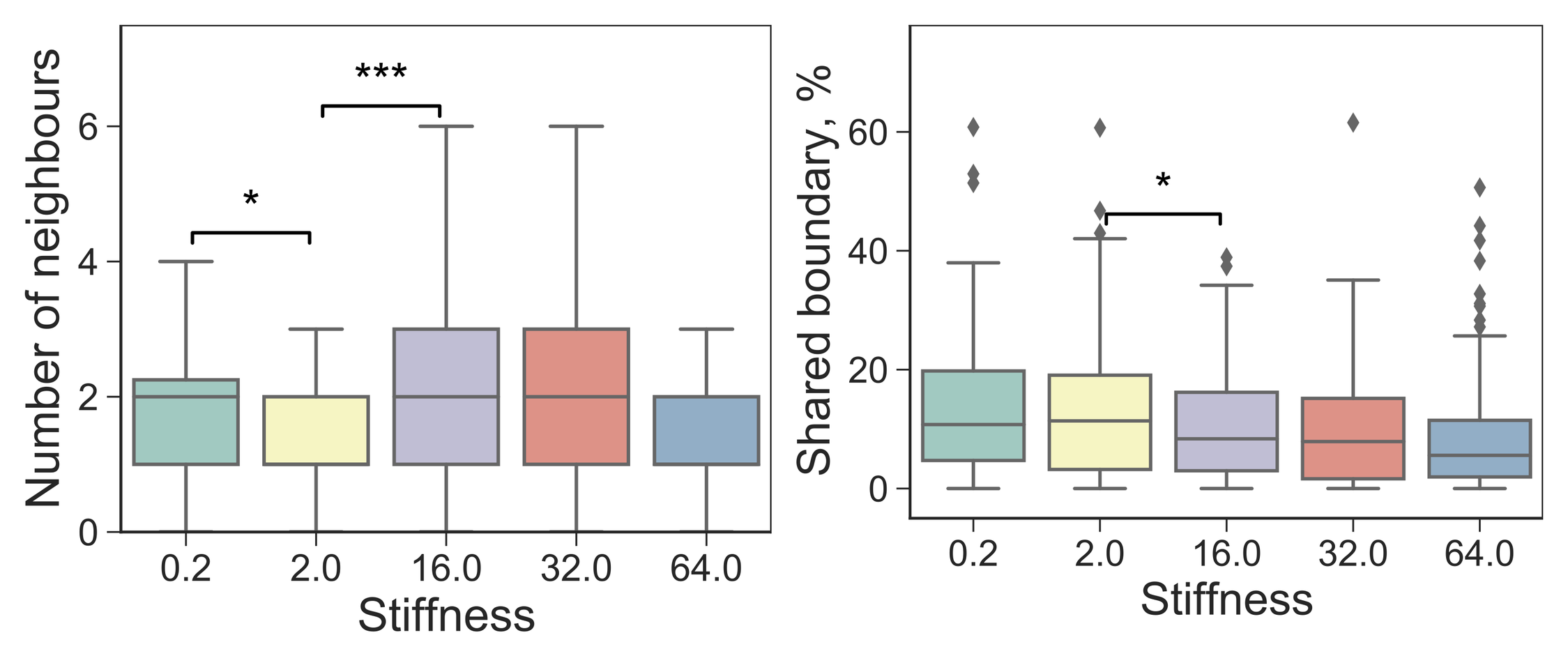


**Fig E.** The number of cell neighbours and the fraction of the shared boundary across stiffness levels.
