# Supplementary material for: Machine learning reveals mesenchymal breast carcinoma cell adaptation in response to matrix stiffness
Source: PLoS Comput Biol. 2021 Jul 23;17(7):e1009193. doi: 10.1371/journal.pcbi.1009193 (PMC8336795; doi:10.1371/journal.pcbi.1009193)
Supplement: S5 Text — (DOCX) [file pcbi.1009193.s005.docx]

# Expression of biomarkers


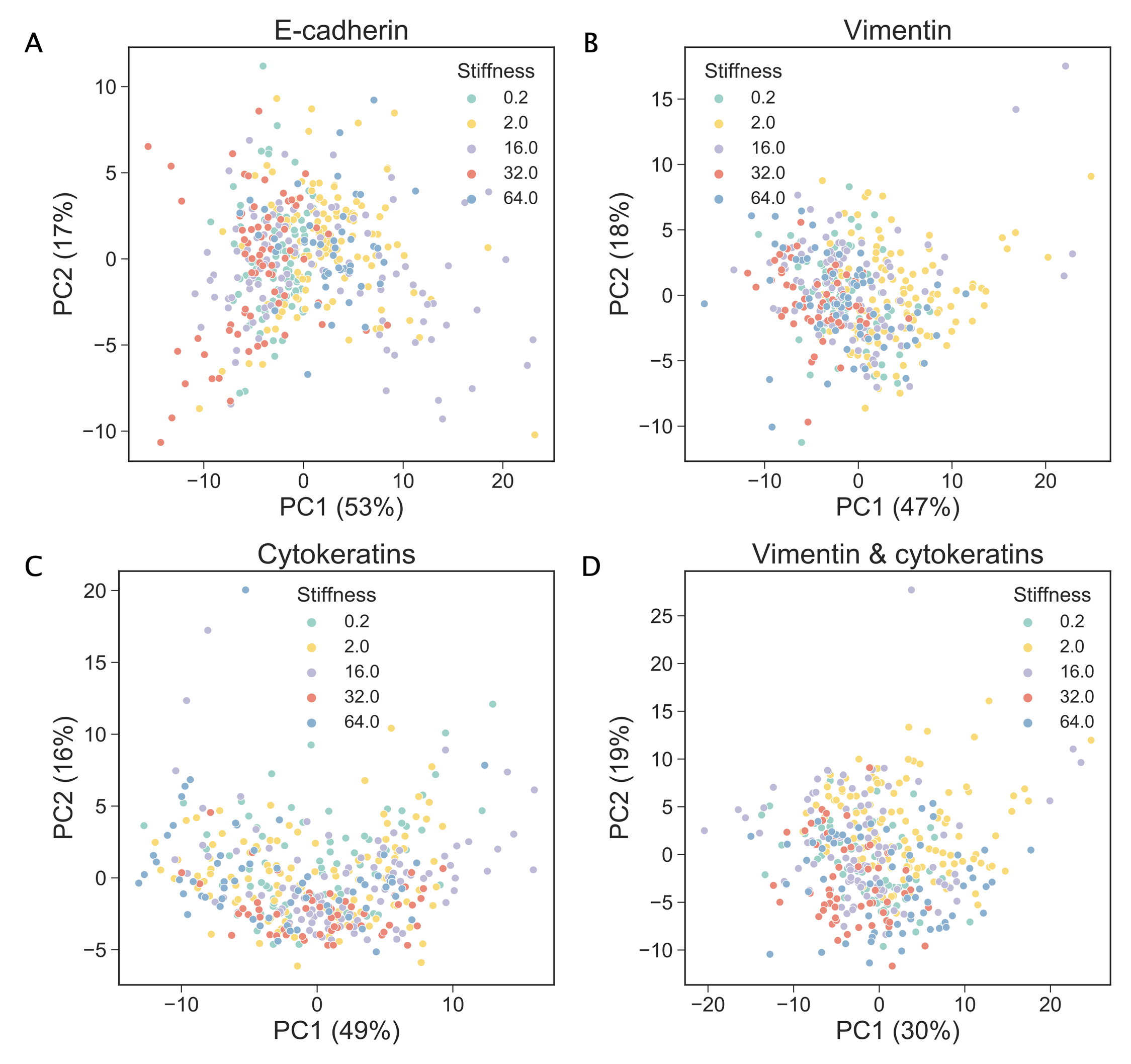


**Fig A.** Results of PCA applied to features describing the intensity and spatial distribution of (A) E-cadherin, (B) vimentin, and (C) cytokeratins. (D) As visualised in Fig 2D and described in the Materials and Methods section, roughly a half of all cells were stained for E-cadherin while the other half was stained for vimentin and cytokeratins. As such, we can also combine the feature sets corresponding to vimentin and cytokeratins.
