# Supplementary material for: Machine learning reveals mesenchymal breast carcinoma cell adaptation in response to matrix stiffness
Source: PLoS Comput Biol. 2021 Jul 23;17(7):e1009193. doi: 10.1371/journal.pcbi.1009193 (PMC8336795; doi:10.1371/journal.pcbi.1009193)
Supplement: S6 Text — (DOCX) [file pcbi.1009193.s006.docx]

# Analysis of multicellular clusters at 64 kPa

## Sensitivity analysis

Among cells cultured at 64 kPa, 84 cells formed multicellular clusters (“Clumped”) whereas the rest appeared as solitary cells (“Single”). Images of cells cultured at 64 kPa were independently examined by authors VSR and ZK and each cell was labelled as being individual or a part of a multicellular cluster. Cells forming such clusters were visibly different and could introduce bias when developing a model aimed to group individual cells thus were excluded from the main dataset and analysed separately.

To perform sensitivity analysis, we included Clumped cells and re-ran the clustering algorithm (Fig A). Inclusion of Clumped cells affected the clustering results by 20% meaning 20% of individual cells (N=168 out of 826 cells) were assigned to a different cell morph. Out of those, N=90 cells were assigned to cell morph 2 instead of cell morph 1. At the same time, the key observation made from Fig 4E remained the same: small round cells (morph 3; blue) were prevailing at 2 kPa with a significant reduction in numbers on the stiffer substrates where cells were predominantly classified as morphs 1 and 2, i.e., stiffer substrates promote cell spreading and elongation with prominent expression of lamellipodia.


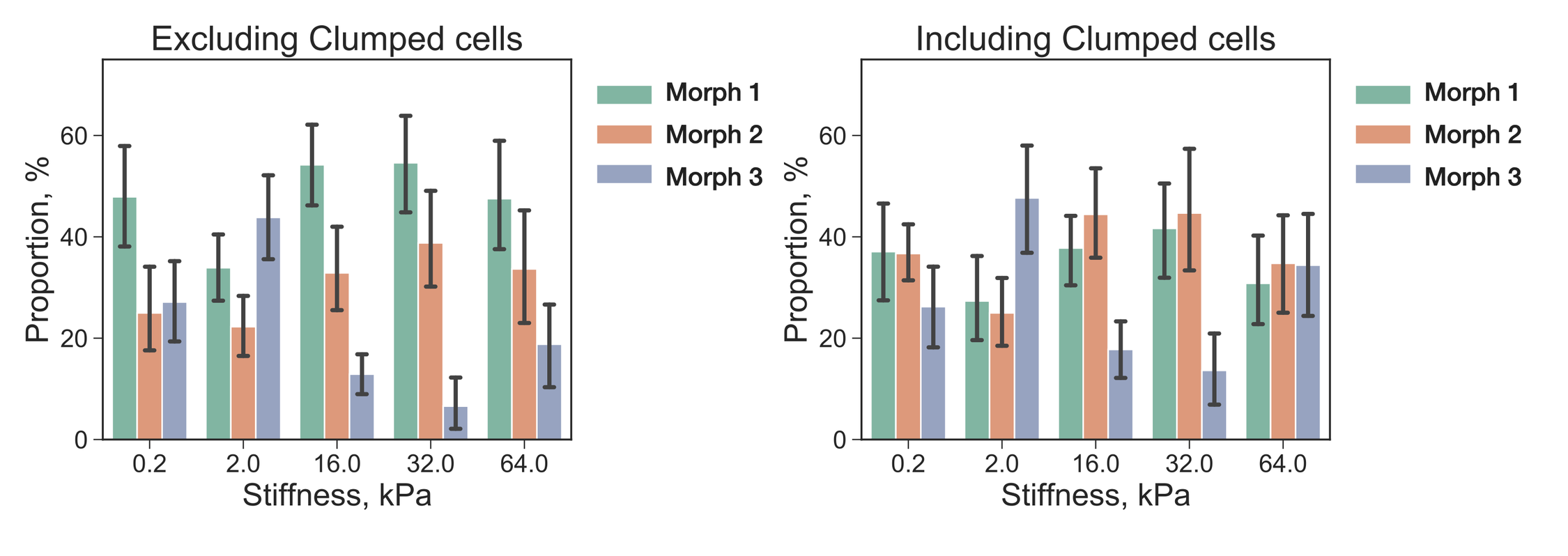


**Fig A.** The proportion of cells of each cell morph across different stiffness levels when Clumped cells are excluded (left; the original Fig 4E) and included (right) for clustering analysis.

## Differences between Single and Clumped cells

To illustrate the differences between Single and Clumped cell populations, we identified features that differed the most between them. For this, we calculated the Pearson’s correlation coefficient (⍴) between each continuous feature and a dichotomous variable “is_clumped” (set to 0 for Single cells and to 1 for Clumped). We ranked features based on the absolute value of ⍴ and selected the top 16 features (with |⍴| > 0.5). To visualise the Single and Clumped cell populations and their average profiles, we standardised each variable to have zero mean and unit variance (Fig B, top). The results below illustrate the differences in the properties between Single and Clumped cells.


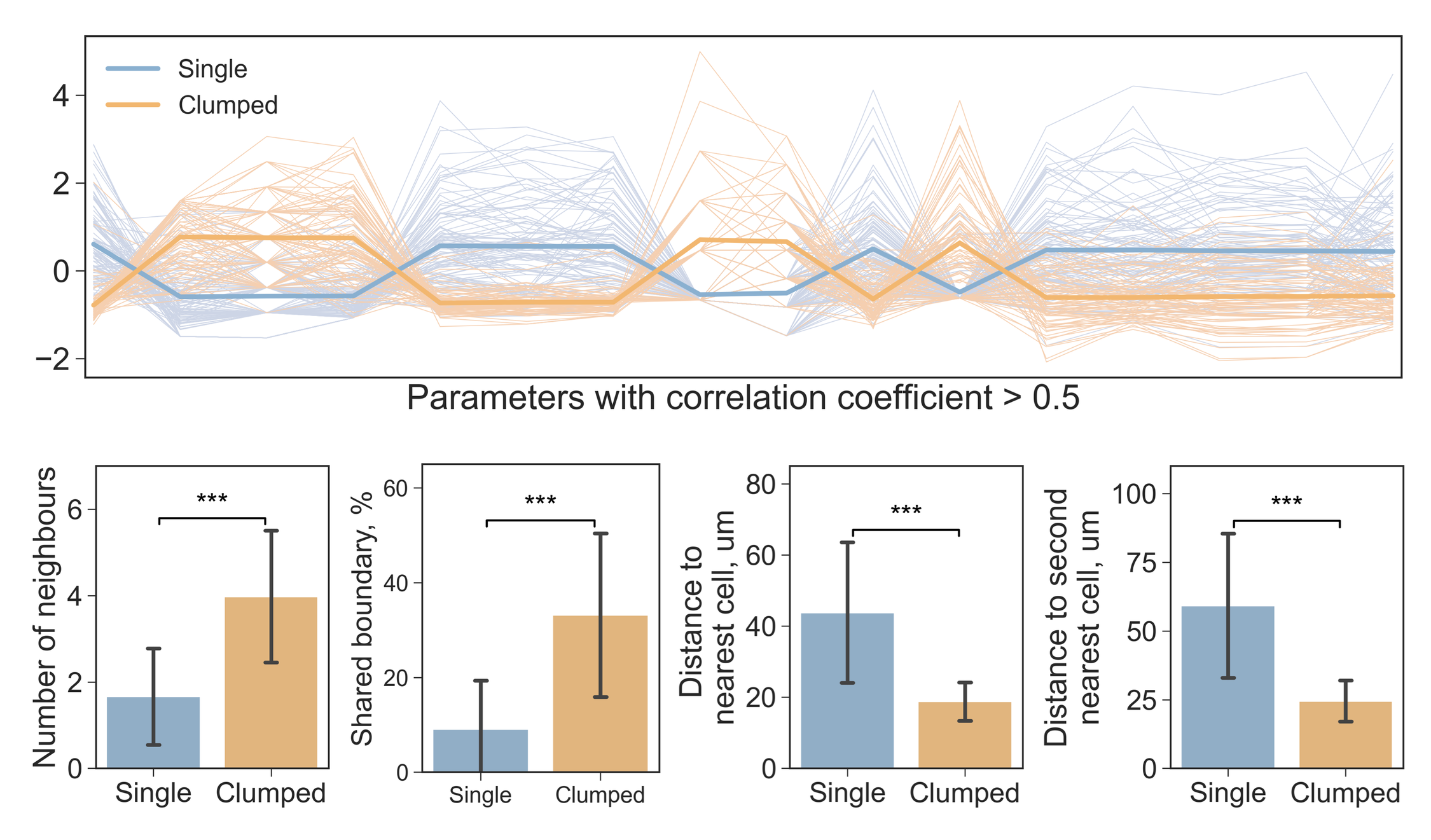


**Fig B.** Top: Correlation analysis revealed parameters in which single cells cultured at 64 kPa differed the most from the clumped cells. Thin lines represent individual cells, thick lines indicate the mean values. Bottom: As expected, the key differences between single and clumped cells were in the context, i.e., numbers of cell neighbours, the fraction of the shared boundary and distances to the nearest cells. Bar plots show means, error bars indicate standard deviation. Significance of the difference assessed by Welch’s t-test (‘***’: p < 0.001).
