# Supplementary material for: Machine learning reveals mesenchymal breast carcinoma cell adaptation in response to matrix stiffness
Source: PLoS Comput Biol. 2021 Jul 23;17(7):e1009193. doi: 10.1371/journal.pcbi.1009193 (PMC8336795; doi:10.1371/journal.pcbi.1009193)
Supplement: S7 Text — (DOCX) [file pcbi.1009193.s007.docx]

# Correlation between ECM stiffness and EMT signature

To test our hypothesis in a clinical scenario and as a proof-of-concept, we analysed genomic alterations (gene expression and copy number variation) of the ECM-signature genes across 1085 breast cancer patients with various types and sub-types from The Cancer Genome Atlas (TCGA) database. Depicted in Fig A, the most genomic variations in expression level of ECM-signature genes and their copy number alterations clustered in patients with Invasive Ductal Carcinoma (IDC) type and TNBC sub-types. Next, we evaluated the correlation between collagen type I (COL1A1) and classical EMT markers VIM, ZEB1, TWIST1 and SNAI2 across 117 patients with TNBC (Fig A). Analysis of Pearson’s correlation coefficient (ρ) confirmed positive correlation between COL1A1 with VIM (ρ = 0.54), SNAI2 (ρ = 0.67), ZEB1 (ρ = 0.68), and TWIST1 (ρ = 0.57). The obtained results from both in-vitro and cohorts analysis indicate role of ECM not only in the induction of EMT and carcinoma cell plasticity but also suggest that ECM-signature genes, particularly expression of collagen-type-I, may serve as indicator of EMT event in tumour microenvironment.


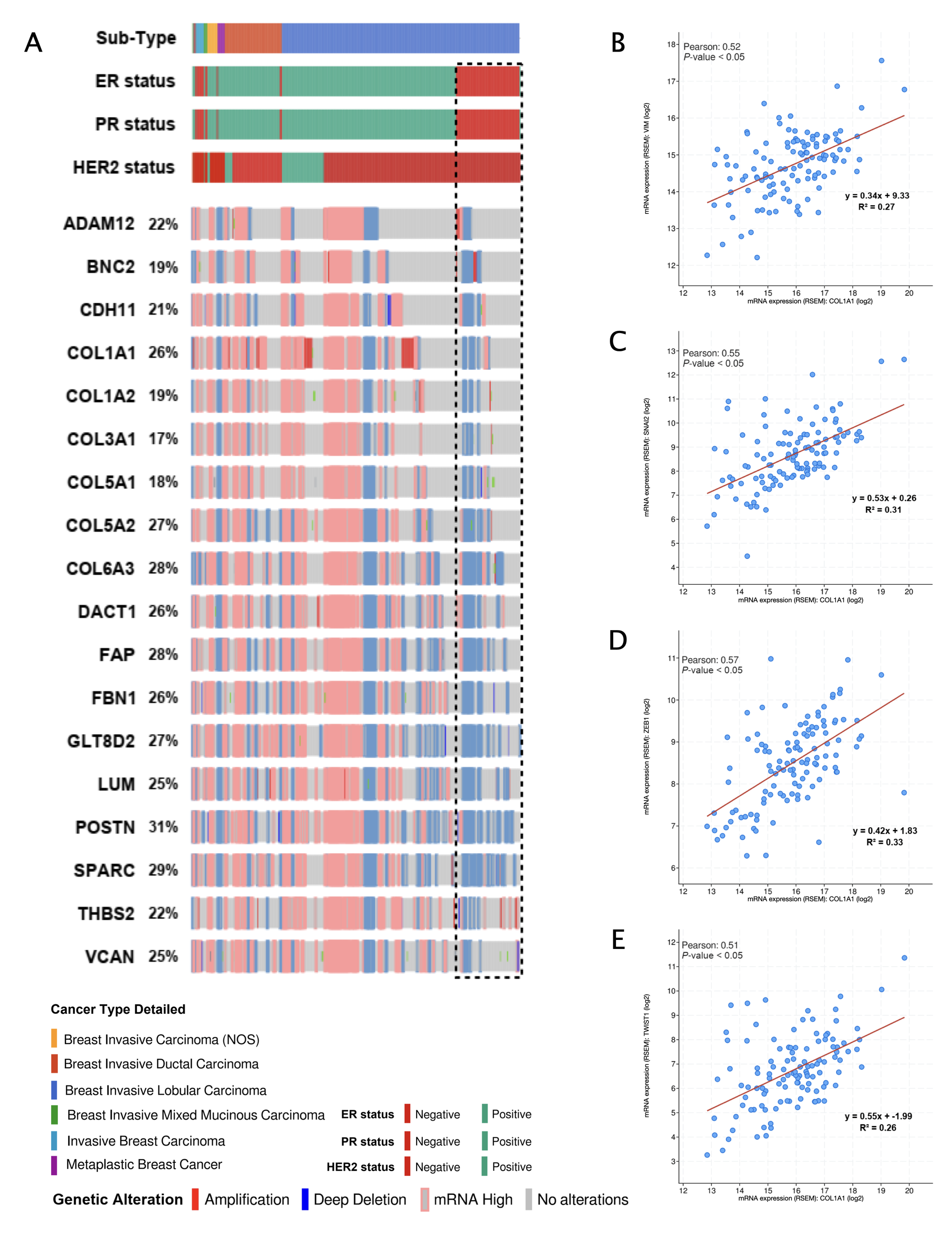


**Fig.** (A) An oncoprint report of the gene expression status and copy number alteration of the ECM-signature genes across 1085 breast cancer patients from the TCGA database. TNBC cases are highlighted by a dashed box. (B-E) Analysis for correlation between collagen type I gene (COL1A1) and selected EMT-related transcription factors VIM (B), SNAI2 (C), ZEB1 (D), TWIST1 (E). Pearson correlation coefficient is reported in the upper left corner, a fitted linear regression model is shown in the bottom right corner of each plot.
