# Supplementary material for: Machine learning reveals mesenchymal breast carcinoma cell adaptation in response to matrix stiffness
Source: PLoS Comput Biol. 2021 Jul 23;17(7):e1009193. doi: 10.1371/journal.pcbi.1009193 (PMC8336795; doi:10.1371/journal.pcbi.1009193)
Supplement: S8 Text — (DOCX) [file pcbi.1009193.s008.docx]

# Fluorescent quantum yield vs microenvironment stiffness

The emission properties of a fluorescent molecule (fluorophore) are measured in terms of a fluorescence quantum yield (QY). In general, QY depends on microenvironment. If the microenvironment properties differ only by the substrate stiffness, the emission properties are affected by optical properties of the substrate. The substrates used in our experiments represent a silicone layer of variable stiffness (Young’s modulus denoted *E*) coated with a thin layer of collagen type I, so that a fluorophore’s interaction with the silicone layer is mediated by collagen type I whose composition is not varied across the substrates. The optical properties of silicone substrates have been studied in literature. For example, Gutierrez et al. designed and tested high-refractive index silicone with variable *E* ranging from to 0.4 kPa to 130 kPa to study cell mobility using total-internal reflection microscopy [PMID: 21961031].  They found that the refractive index of the substrates remained unchanged (1.49).  Commercially available silicone Sylgard 184 with variable *E* is characterised by the refractive index of 1.41 and does not depend on *E*. Therefore, we conjecture that the QY of our fluorescent dyes does not depend on the substrate stiffness.
